# Factors Influencing Water and Sweet Beverage Purchasing Decisions and Behaviours Among Low-Income Households in Four Peri-Urban Communities in Accra: An Exploratory Study

**DOI:** 10.3390/ijerph23060799

**Published:** 2026-06-15

**Authors:** Christopher Delali Amegah, Gloria Adobea Odei Obeng-Amoako, Shu Wen Ng, Monica Lambon-Quayefio, Seth Adu-Afarwuah

**Affiliations:** 1 Department of Nutrition and Food Science, School of Biological Sciences, College of Basic and Applied Sciences, University of Ghana, Legon, Accra P.O. Box LG 134, Ghana; cdamegah@st.ug.edu.gh (C.D.A.); sadu-afarwuah@ug.edu.gh (S.A.-A.); 2 Department of Nutrition, Gillings School of Global Public Health, University of North Carolina, Chapel Hill, NC 27599, USA; shuwen@unc.edu; 3 School of Economics, College of Humanities, University of Ghana, Legon, Accra P.O. Box LG 59, Ghana; mplambon-quayefio@ug.edu.gh

**Keywords:** sweet beverages (SBs), tax, drinking water, bottled water, sachet water, purchasing decisions

## Abstract

**Highlights:**

**Public health relevance—How does this work relate to a public health issue?**

- This study addresses an important public health concern by examining the factors influencing water and sweet beverage purchasing decisions and consumption behaviours among low-income households in four peri-urban communities in Accra, Ghana.
- It provides evidence on how beverage choices are shaped following the introduction of a beverage tax that includes bottled water and fruit juices, particularly in settings with limited access to potable water and among low-income populations.

**Public health significance—Why is this work of significance to public health?**

- The findings show that a majority of low-income households experience water insecurity, with sachet water serving as the primary source of drinking water. Beverage choices are influenced by factors such as taste, convenience, perceived safety, and availability, while sachet water purchases are primarily driven by perceived safety, price, and availability, highlighting the structural determinants of consumption.
- The study further shows that although awareness of the beverage tax was moderate, many participants perceived the taxation of bottled water as unfair and reported substituting taxed sweet beverages with cheaper local alternatives, underscoring potential unintended consequences for health outcomes and health equity.

**Public health implications—What are the key implications or messages for practitioners, policy makers and/or researchers in public health?**

- Limited tax awareness, perceived inequities, and substitution with cheaper sweet beverages highlight the need to refine policy design and strengthen communication to better align fiscal measures with public health goals.
- To strengthen health and equity outcomes, policy makers should consider increasing taxes on sweet beverages to create a clearer price differential between these products and bottled water, while earmarking tax revenues to improve potable water infrastructure and services.

**Abstract:**

Background: In May 2023, Ghana implemented a 20% ad valorem tax on bottled water and sweet beverages (SBs), replacing a 17.5% tax; sachet water remained untaxed. The effect on low-income consumers’ purchasing decisions and consumption patterns remains poorly understood. Objective: We aimed to explore factors influencing water and SB purchasing behaviours among low-income households in four peri-urban Accra communities. Methods: This study employed a convergent parallel mixed-methods design. Four focus group discussions (*n* = 36) and a cross-sectional survey (*n* = 43) were conducted among purposively sampled household primary shoppers in early 2025 across Oyarifa, Teiman, Kweiman, and Danfa. Data were analysed thematically and descriptively. Results: Of 43 participants, 67% were female and 65% had junior high school education. Water insecurity was common (60%), and sachet water was the main drinking source (77%). SB purchasing was driven by taste and convenience, while sachet water choices were linked to perceived safety, price, and availability. Tax awareness was moderate (56%); many perceived bottled water taxation as unfair and reported intentions to switch to cheaper local alternatives. Conclusions: Limited tax awareness and perceived inequities suggest the need for policy refinements to better align fiscal measures with public health objectives.

## 1. Introduction

Poor dietary habits, particularly excessive consumption of sweet beverages (SBs), are a major contributor to the rising global burden of overweight, obesity, and diet-related non-communicable diseases (NCDs) including type 2 diabetes and cardiovascular diseases [1,2]. Globally, NCDs account for 71% of all deaths, approximately 41 million annually with low- and middle-income countries (LMICs) bearing a disproportionate share of this burden [3].

Excessive SB consumption is independently associated with a 26% higher risk of type 2 diabetes, and overweight and obesity are established risk factors for cardiovascular and metabolic NCDs [1]. This burden is driven in part by the concurrent rise of energy-dense, sugar-rich processed foods and beverages alongside persistent undernutrition, a double burden increasingly documented in LMICs [2,3].

At the regional level, the WHO African Region carries 77% of the global NCD burden [4], and sub-Saharan Africa has experienced the most dramatic growth in SB consumption globally: between 1990 and 2018, intake rose by 81.9% to reach a mean of 6.6 servings per week [5]. In Ghana, higher per capita SB sales correlated significantly with a greater number of adults living with type 2 diabetes [6]. Diet-related NCDs accounted for 45% of adult deaths in 2019 in Ghana [4]. The prevalence of diabetes among Ghanaian adults is 6.5% [7], while about half of women aged 20–49 years and one in five men are overweight or obese [8]. Consequently, the World Health Organization (WHO) recommends that countries, including Ghana, implement SB taxation as an effective strategy to reduce excessive consumption and generate public revenue for health interventions [9,10].

Subsequently, Ghana enacted the Excise Duty Act 2014 (Act 878), imposing a 17.5% ad valorem tax on bottled water and non-alcoholic beverages, excluding fruit and vegetable juices [11]. In May 2023, the rate increased to 20% under the Excise Duty Amendment Act 2023 (Act 1093), which expanded the tax to include fruit and vegetable juices. However, Ghana’s tax design undermines the public health intent of the WHO’s SB taxation recommendations, since bottled water is equally taxed, thereby limiting its use as a healthier alternative and substitute for SBs. Instead, it aligns with International Monetary Fund (IMF) recommendations, which primarily aim at generating revenue instead of promoting public health [12].

It remains unclear how Ghana’s beverage tax might lead to price increases passed on to consumers, potentially discouraging excessive SB consumption, as observed in comparable settings such as Mexico [13,14] and South Africa [15,16]. Unlike these settings, since the tax in Ghana also applies to bottled plain water, it is uncertain whether such a design could inadvertently reduce bottled water consumption and prompt substitution with sachet water and other water sources. This is particularly relevant given that access to potable water varies substantially across regions [17], reflecting differences in infrastructure development.

In urban and peri-urban Accra, inconsistent access to potable tap water has led many households to rely on sachet water and other alternatives such as boreholes and tube wells [17]. In urban areas, 52% of households primarily rely on sachet water, 34% on pipe-borne water, and 7% on boreholes or tube wells as their source of drinking water [17]. Although sachet water is widely consumed, its quality, like that of other alternatives, is variable and inadequately regulated.

Low-income households also often face higher exposure to SBs due to economic constraints that make cheaper beverage options more attractive [18]. Price sensitivity exerts a major influence on low-income consumers’ beverage preferences, and budgetary constraints frequently lead to less healthy purchasing behaviour. Moreover, informal markets, which serve as critical access points for low-income communities especially in peri-urban settings, often offer less expensive SBs.

**Figure 1:**
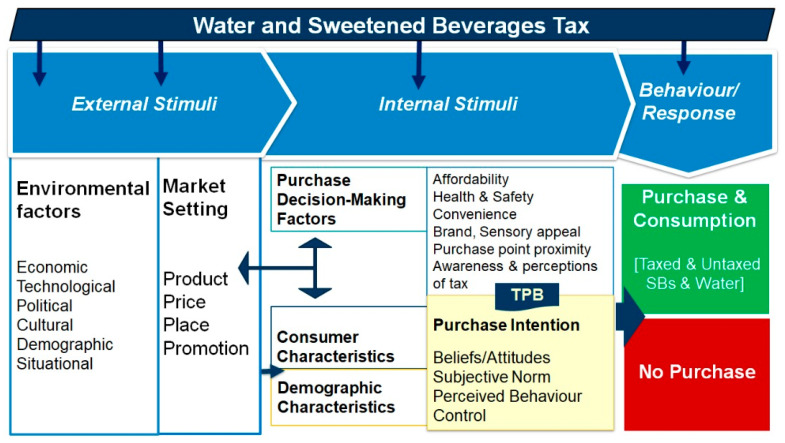
Theoretical framework adapted from the Stimulus-Response Model (Black Box) [19] and the Theory of Planned Behaviour [20].

We adapted a theoretical framework based on the Stimulus-Response Model [19] and Theory of Planned Behaviour (TPB) [20] (Figure 1) as a lens through which we examined how external stimuli interact with internal stimuli to shape purchasing intentions. These internal evaluations shape purchase intentions, which, according to the TPB, are influenced by beliefs and attitudes, subjective norms, and perceived behavioural control. To ease communication, in this study, we used the term sweet beverages (SBs), or beverages, to denote the full set of beverages that contain sugars or other sweeteners, including commercial SBs (carbonated soft drinks, fruit drinks, energy drinks, sports drinks, sweetened milks, and 100% fruit juices) and traditional/local/homemade SBs (such as sobolo, asana, zomkom, and lamugin). Soft drinks refer specifically to carbonated SBs and are therefore a sub-set of commercial SBs. Drinking water sources are reported separately as bottled water, sachet water, piped water, borehole/tube-well water, and other sources.

In this context, low-income households face a dual burden: limited access to potable water constrains healthy hydration choices, while tight budgets heighten sensitivity to the relative prices of taxed and untaxed beverages. Where bottled water and SBs are taxed at the same rate, but cheaper sachet water and untaxed homemade SBs remain widely available, fiscal policy may inadvertently shift consumption toward less regulated alternatives rather than toward water, a pattern with direct implications for the diet-related NCD burden in Ghana. Understanding purchasing decisions under these conditions is therefore central to assessing whether the current tax design supports or undermines its public health intent. We explored the factors influencing water and SB purchasing behaviours among low-income households in four peri-urban communities near Accra. Specifically, we examined (i) household water insecurity experiences, (ii) water and SB purchasing and consumption patterns, (iii) factors shaping purchasing decisions on drinking water (sachet, bottled, and other sources) and SBs, (iv) knowledge, attitudes, and practices regarding SB consumption and health risks, (v) awareness and perceptions of the beverage tax and its benefits, and (vi) how perceptions of the beverage tax influence the purchase of cheaper untaxed homemade SBs among low-income households.

## 2. Materials and Methods

### 2.1. Study Design and Setting

We conducted a community-based exploratory study using a convergent parallel mixed-methods design [21], in which qualitative and quantitative data were collected concurrently, analysed separately using methods appropriate to each strand, and merged at the interpretation stage to produce an integrated account of the factors influencing water and sweet beverage (SB) purchasing among low-income peri-urban households.

The rationale for this design lies in the complementarity of the two strands. Focus group discussion (FGDs) generated context-specific, meaning-rich accounts of how participants reasoned about water and SB purchases. We used a structured household survey to generate frequencies, magnitudes, and category proportions for water and SB purchasing patterns, household water insecurity, and beverage-tax awareness, which are necessary for assessing how widely each driver applies in the study population. Within this design, the household survey served three complementary roles. First, it characterised the socio-economic position of enrolled households using formal indicators like household assets and a derived wealth index, the Multidimensional Poverty Index (MPI), educational attainment, employment status, and household composition, thereby verifying that the housing-based eligibility screen (Section 2.2) had identified low-income households as intended. Second, it produced parallel quantitative measures of constructs that also emerged from the FGDs (primary water source, SB consumption patterns, factors influencing purchasing decisions, and beverage-tax awareness), allowing triangulation of FGD themes with population-level distributions in the study sample. Third, it generated independent quantitative findings that the FGDs were not designed to produce, including the prevalence of household water insecurity using the validated HWISE-12 scale (Section 3.2) and the proportion of participants who would support an SB tax conditional on revenue earmarking (Section 3.7). Convergent integration of the two datasets at the interpretation stage allowed us to identify points of corroboration between the qualitative themes and the survey distributions, points of expansion where one strand added detail the other did not capture, and any points of divergence, which we report transparently. The overall design and the integration of the two strands are summarised in Supplementary File S1, Figure S1.

**Figure 2:**
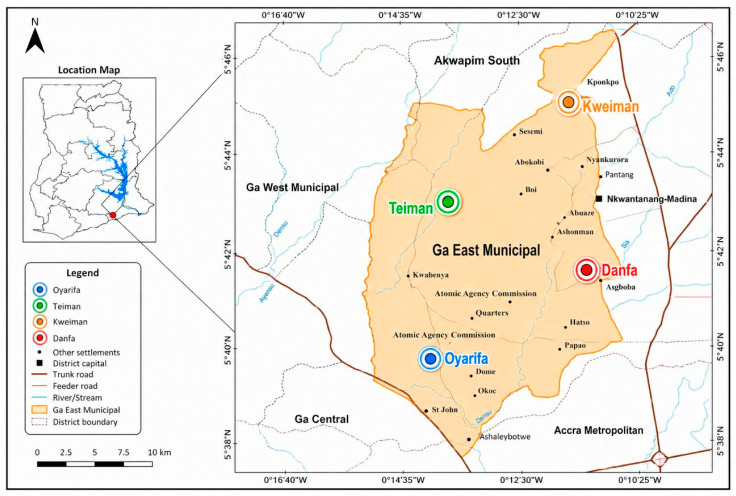
A map of Ga East Municipality showing the four study sites (Teiman, Oyarifa, Danfa and Kweiman).

We collected data between March and April 2025 in four peri-urban communities, namely Oyarifa, Teiman, Kweiman, and Danfa within the Ga East Municipality, located 23 to 49 km north of central Accra (Figure 2). These communities were selected based on their peri-urban character, mixed income profiles, and close proximity to the capital. Peri-urban Accra is experiencing acute infrastructure pressure alongside a profound livelihood transformation, as rapid urban expansion progressively converts agricultural land into residential settlements. Between 2000 and 2010, the agricultural workforce contracted sharply, declining from 19.3% to under 5% [22]. Correspondingly, employment patterns shifted dramatically away from traditional farming toward urban-based economic activities, with wholesale and retail trade emerging as the dominant occupation, engaging approximately 41.9% of the workforce [22].

### 2.2. Study Participants

#### Sampling and Recruitment

The sampling strategy was a multi-stage approach. The sampling strategy operated as a four-stage cascade in which purposive and random selection procedures operated at different stages rather than as competing approaches [23,24,25]. In the first stage, we purposively selected the four study communities because of their peri-urban character, mixed income levels, and accessibility from central Accra. In the second stage, we randomly selected one enumeration area (EA), the standard GSS primary sampling unit, typically containing 100–150 households per community using computer-generated random numbers [8]. In the third stage, we identified eligible households within each selected EA using a random-walk procedure. In the fourth stage, we purposively recruited male and female primary shoppers from eligible households. The full cascade appears in Supplementary File S1, Figure S1.

We screened households against four observable conditions, classifying a household as low-income when it met at least three: (a) walls of mud, wood, metal sheets, or unplastered cement blocks [23,24]; (b) roofs of corrugated iron in poor condition or thatch [23,24]; (c) one or two rooms serving multiple purposes [26]; and (d) structures visibly requiring major repair [23,24,25]. Although this housing-based proxy carries an inherent risk of misclassification, it has been validated for distinguishing low- from higher-income households in West African peri-urban settings without requiring intrusive income questions [25,27,28]. To minimise misclassification, we applied a 3-of-4 rule. At the survey stage, we verified the housing-based screen using formal socio-economic indicators such as household assets, wealth index, MPI, educational attainment, and employment; all 43 enrolled households met the multidimensional poverty threshold (Supplementary File S2), confirming that the proxy screen had identified the intended population.

Finally, we purposively recruited male and female primary shoppers from eligible households. Recruited shoppers worked across both informal and formal employment sectors. Prior to enrolment, each respondent provided oral informed consent. We administered a semi-structured screening questionnaire to an adult member of each selected household, preferably the person responsible for food and beverage purchasing, to identify the primary shopper.

All 43 primary shoppers who completed the household survey subsequently formed the sampling frame for FGD recruitment; 36 (84%) participated in the FGDs, with non-attendance due to scheduling conflicts. Each FGD participant had therefore also completed the survey, linking the qualitative and quantitative datasets at the participant level. Of 54 households screened, 43 qualified for inclusion. The full cascade is depicted in Supplementary File S1, Figure S1.

### 2.3. Data Collection

#### 2.3.1. Quantitative Data Collection

A pre-tested semi-structured questionnaire, adapted from standard questionnaires used in previous studies, was used to collect data on household characteristics and factors influencing water and SB purchasing decisions [15,29]. We assessed household water insecurity using the validated 12-item Household Water Insecurity Experiences (HWISE-12) scale [29]. Data on the frequency of water insecurity experiences covered the four weeks preceding the survey [29]. Other modules covered water and SB purchasing decisions and behaviours, SB consumption (types, frequency, and reasons for choice), perceptions of the beverage tax, and knowledge, attitude, and practice related to SB consumption.

#### 2.3.2. Variables Definitions

Demographic characteristics were described using the following variables: age in years (categorised as 18–35 and 36–64), sex (male, female), marital status (married, single, or widowed/divorced/separated), and religion (Christian, Muslim, or African traditional/other). Educational attainment was classified into five levels: none, primary, junior high, senior high, and tertiary. Household characteristics encompassed household size (≤3, 4, or 5–10 members) and the respondent’s relationship to the household head (self or spouse). We constructed a wealth index using principal component analysis (PCA) based on household assets [30]. Assets included radio or stereo, mobile phone or cell phone, smart phone, television (digital or analogue), refrigerator, deep freezer, computer or laptop, bicycle, motorbike or scooter, car, and truck. We categorised households into tertiles (low, medium, high) to classify socio-economic status. We also assessed household deprivation using the Multidimensional Poverty Index (MPI) based on the Alkire–Foster method [31] (see Supplementary File S2).

We categorised household water insecurity experiences (HWISE) in the last four weeks prior to the survey as never (0), rarely (1–2 times; 1), sometimes (3–10 times; 2), or often/always (11+ times; 3). Summed HWISE-12 scores (ranging from 0 to 36) were grouped into four ordinal categories: no-to-marginal (of 0–2), low (3–11), moderate (12–23), and high (24–36) water insecurity [32].

#### 2.3.3. Focus Group Discussions and Data Collection

##### Guide Development

The FGD guide was developed drawing on both the quantitative questionnaire and the adapted theoretical framework (Figure 1), which outlined the key factors underlying water and SB purchasing decisions and behaviours in the context of the beverage tax among low-income households in formal and informal markets. We pre-tested both the FGD guide and the survey questionnaire (described above) in a pilot study conducted in Bawaleshie, a suburb of Accra, from 29 to 31 March 2025.

##### FGD Participants

All the 43 enrolled primary shoppers completed the quantitative survey. Of these, 36 (84%) subsequently participated in one of the four community-based FGDs, with non-attendance attributed to scheduling conflicts. Each FGD participant was therefore also a survey respondent, ensuring that the qualitative and quantitative datasets are linked at the participant level.

There is no universally agreeable standard for sample size for a qualitative study where FGDs constitute the primary data collection method [33]. Given the homogeneous population across the study communities, four FGDs were conducted. Saturation was operationally defined as the point at which no new themes or issues emerged across successive discussions [34]. Following each FGD, the research team debriefed to assess whether new information was still being generated. By the fourth discussion, recurring themes had been exhausted, and no new issues were raised, supporting the judgement that thematic saturation had been reached.

##### Procedure

Each FGD comprised 8–10 participants and was held at an appropriate venue within the respective community. Sessions were moderated by CDA, an experienced FGD facilitator and a nutrition researcher supported by two field assistants who served as note-takers recording all non-verbal cues and observations relevant to the study.

The guide questions explored a range of issues related to bottled and sachet water and SB purchasing decisions, and behaviours both in informal and formal settings. The topics covered included household water use and purchasing behaviour, SB preferences and decisions, awareness and perception of the beverage tax, reactions to price changes and shaped coping strategies, and perceptions of taxed versus untaxed beverages including trust in the safety and regulation of water and beverages.

Emerging issues identified during the data collection were discussed among CDA, GOA, and the field assistants, and incorporated into subsequent FGD guides. Insights from each site were compared and integrated into subsequent discussions to confirm data or thematic saturation. All FGD sessions were conducted in Twi and Ga, and lasted approximately 90–110 min. We audio-recorded the FGD sessions with participants’ consent.

### 2.4. Data Management and Analysis

We analysed quantitative data with Stata 17.0 software (Stata Corporation, College Station, TX, USA) and used descriptive statistics to summarise continuous and categorical variables.

Two research field assistants transcribed all audio-recorded FGDs verbatim in Twi or Ga and subsequently translated them into English. CDA cross-checked all transcripts to ensure accuracy and completeness.

We analysed qualitative data using a hybrid thematic analysis approach that combined deductive and inductive methods [35,36]. Prior to uploading the transcripts to the software, the research team read all transcripts multiple times and developed preliminary summaries to familiarise themselves with the data and identify potential codes. This immersion in the data informed the development of the initial codebook before formal coding commenced.

CDA and the research assistant uploaded the transcripts to ATLAS.ti 23 (version 25; ATLAS.ti Scientific Software Development GmbH, Berlin, Germany) and coded them independently. In the deductive coding phase, an initial codebook was developed from the study objectives, relevant literature, and the theoretical framework (the Stimulus-Response Model and the Theory of Planned Behaviour), covering drivers such as “price”, “affordability”, and “perceived safety”. In the inductive phase, CDA and a research assistant read the transcripts multiple times to identify emergent themes not captured by the deductive codes, such as “influence of children’s preferences” and “social norms of evening beverage consumption” [37]. During coding, 17 inductively derived codes were added and 30 deductive codes were merged or refined to reflect the data. The final codebook contained 47 codes organised into the six themes reported in Section 3.

The researchers compared coded segments and any disagreements were resolved through consensus-building discussions. This iterative process refined code definitions across all transcripts and confirmed that thematic saturation had been reached. Data saturation was assessed during transcript review and team debriefing; by the fourth FGD, no new major codes or themes were being generated. GOA reviewed the final codes and themes with the research team. 

Coded segments were organised into categories and sub-categories based on their similarities. For instance, codes related to “perceived vendor cleanliness” and “water turbidity” were grouped into the category “perceived safety and health”. From these categories, six overarching themes were generated. To ensure robust themes, findings were synthesised across and within sites and supported with verbatim participant quotes. Site-based matrices and co-occurrence tables were used to compare thematic patterns between communities and to confirm thematic saturation. Quotations that best represented each category were selected for reporting [36].

The final codes and themes were reviewed by the senior researcher (GOA) and discussed with the research team to ensure accuracy of interpretation. We did not seek participants’ feedback on the findings. To maintain transparency and reporting rigour, the study process was documented in accordance with the COREQ 32-item checklist (Consolidated criteria for Reporting Qualitative Research) (Supplementary File S2).

## 3. Results

### 3.1. Socio-Demographic Characteristics of Participants

Nearly 84% of the 43 surveyed participants took part in the FGDs (*n* = 36). The majority of FGD participants were female (67%), and just over half (56%) were between 18 and 35 years old. Most participants in both the survey and FGDs identified as Christians (88.4% and 88.9%, respectively). Nearly half were both the household head and the primary shopper responsible for purchasing sachet water and SBs. The majority had attained a junior high school level of education. About one-quarter of the surveyed participants lived in households classified within the lowest wealth index (Table 1). Most households lived in houses with cement block walls and metal roofing. Only 10 of the 43 households owned flush toilets (Supplementary File S4: Additional Results Table S1).

Six main themes emerged from the qualitative data. The main themes included: (1) household water insecurity, (2) water and SB purchasing and consumption, (3) factors influencing water and SB purchasing decisions and consumption, (4) knowledge, attitudes, and practices related to SB consumption and health risk, (5) awareness and perceptions of the beverage tax and its benefits and (6) perceived impact of the beverage tax on patronage. Several sub-themes were identified within each theme.

### 3.2. Household Water Insecurity Experiences

Over half of the households surveyed (60%, *n* = 26/43) reported experiencing water insecurity in the four weeks preceding the study, comprising low (20.9%), moderate (23.3%), and high (16.3%) water insecurity (Table 2). The Teiman community recorded the highest rate of water insecurity compared with the other study sites (Supplementary File S2: Figure S2). These survey findings were corroborated by FGD accounts, in which participants attributed water insecurity primarily to irregular pipe water supply, with some households reporting receiving piped water only twice a month. Participants reported that irregular supply made it difficult for households to meet basic needs such as drinking, cooking, bathing, and washing and reported routinely rationing or drawing on multiple sources (sachet, borehole, stored, and piped water) to cope.

“The pipe water is not available all the time. Sometimes, when they open the pipe water twice in a month. If it were consistent, the pipe water would come more often, and if we do not have the money to buy sachet water, we could just drink pipe water. I think that would be better for us than relying on borehole water.” (Person 7, Female, Danfa).

“…the cost of water has really gone up. For over a year now, the pipe has not been flowing. The polytank water we buy is from people who set their own prices based on how much they paid. That’s why it’s so expensive now” (Person 3, Female, Oyarifa).

“…If your household is large and you need to buy four or five bags of sachet water, sometimes you simply can’t afford it. The price is scary, so if your family is big, you can’t buy [sachet water] often.” (Person 9, Female, Teiman).

“One bucket of water costs GHS 1.00…not enough for my young daughter and me to bathe. But we use a lot of water, and not just for bathing but also for cooking and other things, and that’s why the cost is high.” (Person 10, Female, Teiman).

While the survey established the prevalence and severity of water insecurity, the FGD accounts illuminated how participants linked irregular supply and rising costs with their daily coping strategies, particularly among larger households and those in the Teiman community. Coping strategies were not captured in the survey; this dimension was contributed entirely by the qualitative strand.

### 3.3. Water and Sweet Beverage Purchasing Behaviour and Consumption

Sachet water, also known as pure water, was the primary source of drinking water among the surveyed participants (79.7%). More than half of the households purchased at least three bags/packs (i.e., 45 litres of water) weekly, with a third spending over GHS 50.0 on sachet water per week (Table 2). Regarding SB consumption, the majority of the households (69.8%) reported consuming commercial SBs, predominantly carbonated varieties, frequently. Nearly two-thirds purchased them at least daily or three to four times per week and over a third spent more than GHS 50 on SBs weekly (Table 2). The survey findings were broadly consistent with FGD accounts, in which participants described regular consumption of both commercial SBs such as Bel Cola, Bigoo, and Malta Guinness and traditional/local SBs or homemade SBs such as sobolo (made from hibiscus leaves), asana (made from malted maize), and zomkom (made from rice). The FGDs added detail that the survey could not capture, revealing the specific brands consumed, the role of traditional SBs as everyday alternatives and the social context in which both types of SBs were consumed.

“For me, I buy Bel-Cola. They sell it for GHS 4.00 at the store. I take three bottles in a day.” (Person 1, Female, Danfa).

“For me, I buy asana (traditional beverage) from a street vendor, it costs GHS 2.00. If you have GHS 2.00, you can get one to drink, so for me when they are passing, I buy it.” (Person 3, Female, Danfa).

“For me too, sometimes I buy rice to make a traditional beverage …called ‘zomkom’…I make zomkom for myself and my children to drink.” (Person 9, Female, Danfa).

While the survey quantified the frequency and expenditure patterns of SB and sachet water purchasing, the FGD accounts extended this by illustrating the variety of beverages consumed across commercial and traditional categories, and by capturing the everyday economic trade-offs households made between them.

### 3.4. Factors Influencing Water and SB Purchasing Decisions and Consumption

#### 3.4.1. Factors Influencing Water Purchasing and Consumption

**Figure 3:**
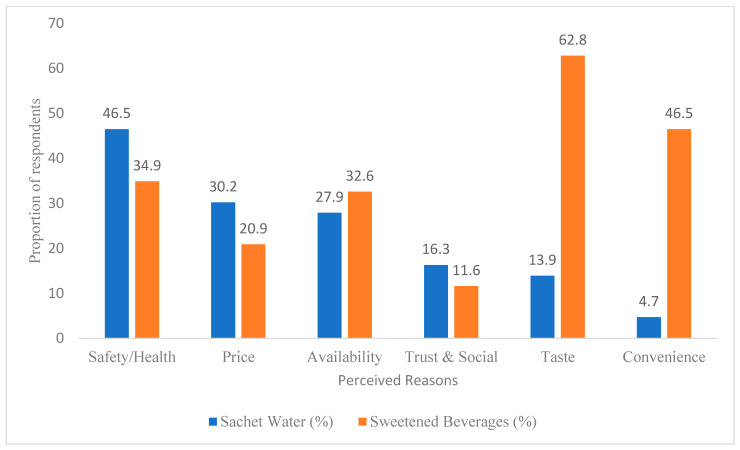
Factors influencing water and SB purchasing and consumption among low-income households, March–April 2025.

When surveyed participants were asked to mention the three most important factors influencing their choice of drinking water, they reported perceived safety and health (46.5%), price (30.2%), availability (27.9%), trust and social factors (16.3%), taste (13.9%) and convenience (4.7%) (Figure 3; see Supplementary File S4: Tables S2–S4 for additional results). The FGD findings were broadly consistent with these survey rankings and provided additional depth on why these factors shaped participants’ purchasing decisions.

Perceived safety and health: A majority of participants reported that cleanliness and perceived safety considerations shaped their choice of sachet water brand. In this context, “perceived safety and health” refers principally to participants’ assessments of microbial cleanliness, vendor hygiene, brand trustworthiness, and prior experience of illness, rather than to long-term diet-related health. Many reported that they visually inspected the water, checking for the absence of turbidity or impurities before purchasing sachet water:

“I check the cleanliness of the water, whether the water is clear before I buy.” (P1, Female, Teiman).

For safety and infection prevention, most participants preferred to consistently purchase one particular brand of sachet water.

“At first, when I got very thirsty, I would drink any water I found. Later, I fell sick…and I was diagnosed with typhoid. The doctor advised me to stop drinking random sachet… So now, I drink Clean Pack (sachet water brand).” (Person 6, Female, Oyarifa).

“My child fell ill…the doctors advised us to drink only one type of water and not mix [brand], since then, we’ve all been careful.” (P2, Male, Kweiman).

Some participants reported checking expiry dates and looking for Food and Drug Authority (FDA) endorsement when purchasing water. Although sachet water was generally trusted, nine households reported episodes of stomach pain or diarrhoea after drinking sachet water (Supplementary File S4: Table S2). This finding from the survey was echoed in the FGDs, where participants described illness experiences as closely linked to brand loyalty, a dimension the survey rankings alone could not capture.

“I check the expiry date. For water, the label usually says it expires in three months.” (Person 5, Female, Teiman).

Price: A majority of the participants reported they preferred sachet water to bottled water because of the perceived high cost of bottled water. While bottled water was often regarded as safer and of superior quality, it was considered too costly for regular household consumption. This is consistent with the survey finding that price was the second most cited factor (30.2%). Some participants found sachet water cheaper and thought that bottled water was same as sachet water. One participant remarked,

“Do you know something?… Dede and Korkor are both Ga children” (Person 5, Male, Danfa).

This statement highlights the perceived similarity between bottled and sachet water, like how children from the same Ga family or tribe are the same, implying sachet water and bottled water both contain water. So, purchasing sachet water was considered economically sensible, as it provided sufficient quantities for the entire household as one participant explained:

“I would love to buy five bottles every day, but instead, I can buy a whole pack of sachet water for my children and myself. Bottled water is of better quality than sachet water, but the price is too high.” (Person 9, Female, Teiman).

Participants emphasised that sachet water was the most preferred water for drinking because of its affordability. The option of purchasing sachet water in bulk at a wholesale price was described by participants as helping them stretch limited budgets:

“With sachet water, a bag/pack costs GHS 6.00 when purchased wholesale, but individually (retail price) it is GHS 10.00. Bottled water is far more expensive. Personally, if I’m using my money, I hardly ever choose the bottle unless it’s absolutely necessary.” (Person 2, Male, Kweiman).

Availability: Beyond affordability, sachet water was also widely consumed because of its availability and accessibility. This aligns with the survey finding that availability was the third most cited factor (27.9%) for purchasing decision. Participants repeatedly described sachet water as being “everywhere”—on streets, in kiosks, or delivered directly to households by motor tricycles, locally known as aboboyaa.

“Sachet water is everywhere. You can get it on any street corner. But with bottled water, you usually only see it at events like weddings, naming ceremonies, or funerals.” (Person 4, Male, Teiman).

“…Some vendors bring it [sachet water] to us using motor tricycles” (Person 8, Male, Oyarifa).

“Here [in this community], wherever we buy our water, it’s sachet water that we buy… wholesale…It’s loaded onto a tricycle [“aboboyaa”]…” (Person 2, Male, Danfa).

Trust and social factors: Participants mentioned that trust in the source or vendor was linked to their choice of where to purchase water. Vendor cleanliness and proper handling and storage of sachet water were cited as important considerations. Many participants reported buying sachet water from neighbourhood kiosks, or small local stores or shops whose owners they had established relationships with or had family ties to.

“There are a lot of places that sell sachet water…in my area here. I prefer to buy from my sister-in-law; she is a family member, that is why I buy from her or I buy from someone I am in good terms with. I don’t buy from just anyone.” (Person 9, Female, Kweiman).

“I buy mine from nearby shops because it’s close to home” (Person 10, Female, Teiman).

“As for me, my wife’s father sells water and drinks, so sometimes when there is none at home, I just call him and he brings some for me” (Person 4, Male, Oyarifa).

“…At times, sachet water is stored directly on the floor, and I believe this can also affect the water. Water can become bad because of how it is stored…” (Person 2, Male, Kweiman).

Taste: Participants also mentioned the taste of water as a consideration in their purchasing decisions. Some expressed concerns about offensive odour and unpleasant aftertastes in certain water brands. Others disliked a salty taste or suspected the presence of chemicals. Some participants noted that chilling improved the taste and made it easier to drink. Participants also revealed that storage conditions were associated with taste; however, some brands were preferred because water remained consistently tasty and fresh even after prolonged storage.

“…it tasted like normal water like actual water. But… now, when you drink it, it tastes more like well water…if it’s not chilled, you can’t drink it. But even if it’s slightly chilled, you can manage to drink a little because of the chemicals or substances they’ve put in it, it doesn’t taste good anymore” (Person 4, Male, Kweiman).

“If you buy and store it, it doesn’t change taste or smell, it stays the same. Even if you store four bags/packs, you know it will remain clean and fresh with no scent change. That is why we buy it. Even if you store it, the scent does not change…so that is what is good” (Person 6 Female, Oyarifa).

Convenience: Participants highlighted several practical considerations that made sachet water their preferred choice for households. Sachet water could be purchased in small amounts daily especially when chilled or in bulk depending on available funds, and it was easily transported by vendors using tricycles. Participants also noted that bottled water, while re-sealable, was not always practical for children; sachets were easier to share or buy individually for school. However, sachets often spilled, and some respondents felt they were less hygienic to handle compared to bottles. Despite these limitations, sachet water remained the popular choice of drinking water for daily consumption.

“If you’re a parent moving (traveling) with a child, it’s better to use bottled water. With sachet water, I can drink all at a go, but my child might not be able to drink all at once, and the rest might spill. You can’t keep it in your bag neither because it’ll spill and wet your stuff, but with bottled water, they can drink some and close it for later. So, for such situations, bottled water is the better choice.” (Person 2, Male, Kweiman).

“Although, pouring sachet water into the bottle will fill it up, there’s still a difference… But when my child is going to school and I don’t have enough money to buy bottled water, since my child has a bottle, I’d rather fill the bottle with sachet water.” (Person 6, Female, Kweiman).

Both survey rankings and FGD accounts were consistent in identifying perceived safety and price as the most commonly cited factors in water purchasing, while the FGDs added expansion through accounts of brand loyalty, illness-driven brand choice, and the social significance of bottled water at events. No substantive divergence between the survey and FGD data was observed for water purchasing.

#### 3.4.2. Factors Influencing SB Purchasing Decisions and Consumption

Although participants identified similar reasons as with drinking water purchases, the relative importance of the factors influencing SB, including local drinks/homemade drinks, purchasing differed (Figure 3). Taste (62.8%) was the most cited important factor, followed by convenience (46.5%), perceived safety and health (34.9%), availability (32.6%), and price (20.9%) among the survey participants. Participants also considered trust and social factors (11.6%) (Figure 3) in their decisions. FGDs provided deeper insights into the reasons underlying SB purchasing decisions.

Taste: Taste was the most important factor associated with beverage choices. Participants noted that the sweetness of SBs motivated both their purchase and consumption, often prioritising sensory appeal over price. Participants described regular consumption of SBs as associated with taste preferences:

“It depends on the taste you prefer in your drink. That’s why I say I don’t think the price change has really affected the consumption.” (Person 2, Male, Kweiman).

“Some people drink it simply because it tastes good to them.” (Person 9, Female, Teiman).

“Have you seen…mostly the thing we look at is the sweetness…everyone with their own tongue (taste buds/preferences)…and so when you drink and taste the sweetness…” (Person 5, Male, Kweiman).

Convenience: The convenience of delivery, widespread availability, and access to “chilled” SBs were cited by participants as associated with their purchase and consumption patterns. Households valued accessing SBs through tricycle (aboboyaa) delivery, nearby kiosks, or vendors with reliable refrigeration. Cold SBs were particularly preferred during hot weather to “cool their hearts”.

“We buy from the car or the store, but mostly the aboboyaa comes to the house.” (Person 1, Female, Teiman).

“If the sun is scorching, you have to watch and see whose fridge is working, so you can buy chilled water or drinks that can cool your heart. That’s why we buy from where we like.” (Person 7, Female, Kweiman).

“Around 1 or 2 in the afternoon when the sun is really hot and I’m feeling warm, I just reach home and I’m sweating, I call my child “child please take ‘Bel Cola’ from the fridge. I like the ones that are a bit chilled…and cool my heart.” (Person 10, Female, Kweiman).

Price: Price was cited as a major consideration, with households often opting for cheaper brands or smaller quantities. Many participants considered local SBs, such as sobolo or asana, as more affordable alternatives to commercially produced beverages.

“Nowadays, commercial SBs are for those who can afford them. If you can’t, you make your own sobolo at home” (Person 8, Female, Danfa).

Brand familiarity and loyalty: Participants mentioned their habitual purchase was attributed to long-standing brand familiarity and loyalty.

“Oh, in the past when I had money, I used to drink ‘Malt’. But these days, since I don’t have money, I go for ‘Bigoo’ or ‘Bel Cola’.” (Person 9, Female, Teiman).

Social norms: Participants reported their consumption of SBs was influenced by social practices. Participants reported they purchased SBs because they observed others doing so. Regular SB consumption in the evenings after meals was described as a common household norm:

“…In the evening after eating when you feel thirsty, you can take some and pour it over ice. So, we should have some drinks at home” (Person 1, Female, Danfa).

Cravings: Beyond economic factors, impulsive cravings for sweetness emerged as a powerful driver. Participants described an urge to satisfy these yearnings that often led to purchasing larger quantities than originally planned.

“…sometimes you are just craving it and need something to drink. You may end up buying two or even more without planning to…” (Person 1, Male, Oyarifa).

“One, they are affordable. Two, they are easily accessible. Three, sometimes you just crave them” (Person 5, Male, Oyarifa).

Children’s preferences: The presence of children in households significantly shapes SB purchasing and consumption patterns. It is a common practice for parents to maintain a stock of beverages at home to provide as snacks or for children to take to school. Participants emphasised that children’s specific tastes often dictate which brands are purchased, frequently overriding parental choice. When faced with rising prices, parents often employ an economic substitution strategy, switching to cheaper commercial brands or traditional local alternatives like sobolo for their children rather than reducing consumption. Participants’ accounts suggest that children’s preferences are linked to higher household consumption, particularly when SBs are easily accessible through nearby informal vendors.

“Because of the children, there should be some drinks at home…” (Person 1, Female, Danfa).

“Before, it was ‘Squeeze’ that we used to buy for the children, but now, we buy drinks like Bel-Cola and Coke for them…Now it’s only Bel-Cola. We go for the cheaper one” (Person 2, Male, Danfa).

“For the children, when they go to school, we sometimes buy them sobolo or other local drinks, because the bottled ones are too expensive” (Person 5, Male, Oyarifa).

“If the drink is expensive, we don’t buy it for our children. What we look for is what the children like” (Person 5, Male, Oyarifa).

“You can’t dictate to the children what to drink. Maybe you will buy Bel Cola but the child will say he/she prefers Puka, you must buy what each prefers so whatever each person prefers, that’s what you end up buying and drinking throughout the week” (Person 3, Female, Oyarifa).

Survey rankings and FGD accounts again converged on taste and convenience as the dominant SB purchasing drivers, with the FGDs adding expansion on cravings, brand loyalty, and children’s preferences. The relative importance of price differed modestly between the two strands; price was ranked fifth in the survey but featured prominently in FGDs as trade-offs between commercial and traditional SBs, a pattern best interpreted as an expansion rather than a divergence.

Perceived safety and health concerns: Participants reported that concerns about health consequences of excessive intake of SBs influenced purchasing decisions and consumption. Some participants said they limited intake of SBs because of the perceived high sugar content, artificial additives, and the risk of long-term health effects. As a result, participants reported diluting drinks with water, reducing frequency of purchase, or avoiding certain brands altogether.

“Before, when you bought Coke, you could taste the cola in it. But now when you buy Coke, everything tastes like sugar, you don’t taste the cola…So I plead that they should reduce the sugar in these drinks because it’s not good for us!” (Person 2, Male, Danfa).

“I don’t really buy any (drinks). I believe they are just made of sugar and coloring…I prefer to buy sobolo leaves and make it at home.” (Person 2, Female, Teiman).

Some participants expressed concerns about how SBs were produced, handled, and stored, suspected the use of chemical additives, and doubted hygienic practices, particularly the street-vended SBs.

“If you ask me, the local drinks are better. The commercial ones (SBs) are full of chemicals.” (Person 5, Male, Oyarifa).

“Some of the street drinks can be risky, but what choice do we have?” (Person 4, Male, Oyarifa).

These accounts indicate that awareness of health effects was associated with SB purchasing decisions in this population, but unevenly. Where participants linked a specific SB to a tangible health concern (such as perceived excessive sweetness, suspected chemical additives, or hygiene of street-vended drinks), they reported concrete adjustments such as reducing intake, diluting drinks, switching brands, or preparing traditional alternatives at home. Where awareness was lower, as with the sugar content of energy drinks, fruit juices, and traditional beverages (Section 3.6), purchasing patterns were less responsive to health considerations. The data therefore suggest that consumer health awareness was selectively associated with SB purchasing, and that broadening awareness beyond diabetes risk may be necessary to support reductions in consumption.

### 3.5. Knowledge, Attitudes, and Practices (KAPs) Related to SB Consumption and Health Risks

When participants were asked to mention what came to their minds when they thought of the term “sweetened beverages”, soft drinks (e.g., Coca Cola, Sprite, carbonated drinks) were the most frequently mentioned (*n* = 42/43, 97.7%), followed by nectar/canned juices (*n* = 13/43, 30.2). Only 11 (25.6%) participants mentioned traditional drinks. Overall, participants demonstrated generally low knowledge of SBs (90.7%) (Supplementary File S4, Table S5). These survey findings indicate that participants’ understanding of what constitutes an SB was largely limited to carbonated SBs, with limited recognition of traditional SBs, juices, and other sweetened products as SBs.

Table S6 in Supplementary File S4 shows the perception of sugar content in beverages. All participants correctly identified still water as “not sugary”; however, only 37.2% perceived energy drinks as “somewhat sugary”. Participants viewed homemade SBs (65.1%) and locally made SBs (58.1%) as “somewhat sugary.” Knowledge of powdered drinks, energy drinks, diet sodas, and cordials as SBs was limited (Supplementary File S4, Table S6). These findings suggest that participants tended to underestimate the sugar content of energy drinks and locally made beverages, while correctly identifying commercial carbonated SBs as sugary, a pattern consistent with selective risk percention.

Participants perceived that SB consumption increased the risk of diabetes (83.7%) while a majority (81.4%) did not perceive a link between SB consumption and cancer. Just over half (53.5%) did not perceive a link between SB and obesity (Supplementary File S4, Table S6). These findings point to a selective pattern of health risk awareness in which diabetes was widely recognised as associated with SB consumption, while links to obesity and cancer were less commonly perceived. This pattern was largely captured by the survey: the KAP data were primarily quantitative in this section, with the FGD accounts in Section 3.4.2 providing qualitative depth on health awareness being associated with purchasing behaviour in practice.

### 3.6. Awareness and Perceptions of the Beverage Tax and Its Benefits

Although just over half of surveyed participants (55.8%) reported some level of awareness of the beverage-tax policy, 44.2% had no knowledge of it (Table 3). The FGD accounts were consistent with this survey finding, indicating that even among those who reported awareness, understanding of the tax was limited. Participants frequently conflated general price increases driven by inflation with those specifically attributable to the tax, and many believed the tax was levied twice—once on producers and again on consumers—creating a perceived sense of double burden.

“Sometimes you’ll go to buy it and it’s GHS 3.00, but they’ll tell you GHS 5.00 next time and it’s because of the added charges (taxes), so we want to know how much exactly has been added.” (P3, Female, Oyarifa).

“The tax is two ooh, they [government] tax the producer and then me…the consumer too, why tax me?” (Person 5, Male, Oyarifa).

These quotes illustrated participants’ confusion about price changes and their perception of unfair double taxation. Participants expressed strong opposition to the taxation of bottled water, arguing that water is a basic necessity for survival and should not be treated as a taxable commodity.

“Water is life, so they [government] should do something about it for us…Because sometimes, when you’re very thirsty and you have only 50 pesewas, you can’t even buy water. That’s a big problem.” (Person 10, Female, Oyarifa).

“For me, water is essential to life. So, if they could reduce the price, it would help us. Because sometimes, if we don’t even have food…water alone can help us survive” (Person 2, Female, Teiman).

“Water is like air. Just as we need air to live, we also need water. In past years, we used to buy a sachet for 10 pesewas and a pack for GHC 2.00 or 3.00. But…now, a pack costs around GHC 6.00 or even more” (Person 1, Female, Teiman).

Participants further argued that other goods were more appropriate targets for taxation than water:

“There are many other important things we could use taxes for. If there is a tax on water, I won’t support it, because water is essential and we already pay too much. There are better things to tax” (Person 1, Female, Teiman).

These sentiments were reflected in the survey findings: more participants opposed the taxation of bottled water than taxation of SBs (62.8% vs. 46.5%), a pattern consistent with participants’ perception that water was perceived as a fundamental necessity for human survival and should be affordable and universally accessible. The survey proportions on opposition to water taxation were borne out by the qualitative accounts, in which participants described water as a necessity that should not be taxed. Notably, however, when asked whether they would support an SB tax if the revenue was earmarked specifically for improving water infrastructure, the majority (79.1%) indicated support (Table 3).

### 3.7. Perceived Impact of the Beverage Tax on Purchasing of Cheaper SBs

Participants’ perceptions of the tax’s impact on their SB consumption were mixed. While a majority (69.8%) indicated that they would continue to consume the same SBs regardless of the tax, others reported that they would reduce their consumption (44.2%) or switch to untaxed or locally produced SBs (46.5%). The most commonly mentioned untaxed local/traditional SBs were sobolo (made from hibiscus leaves), asana (a malted corn drink), and lamugin (a millet-based drink). The FGD accounts were broadly consistent with these survey findings and provided additional depth by illustrating three distinct response patterns among participants. First, some of the participant reported that as prices rose, they bought local SBs more often:

“The price of drinks keeps going up, so we just buy the local ones now. Even for the kids.” (Person 2, Male, Danfa).

“If you can’t [afford commercial SBs], you make your own sobolo at home.” (Person 8, Female, Kweiman).

Second, others described purchasing decisions as entirely determined by available funds on any given day, moving fluidly between commercial (taxed) and traditional (untaxed) SBs depending on what they could afford:

“Everything depends on the person’s pocket. If someone has enough [money], they’ll still buy the ones they used to buy. But if not, they’ll switch to the local ones…so, we will buy the local ones more” (Person 3, Female, Oyarifa).

“…Maybe today, I won’t even have GHS 4.00 to buy the drink [commercial SB]. But maybe I have GHS 2.00 and I buy my sobolo [traditional SB]…that’s okay with me” (Person 6, Female, Oyarifa).

Third, a number of participants indicated that strong taste preferences and ingrained habits were described as associated with continued SB consumption regardless of price increases. Sweetened beverages were described as ingrained in daily routines and social enjoyment:

“Even if they sell it at GHS 1000, we will still buy it” (Person 8, Female, Teiman).

“You can still buy the taxed ones if you want…it’s only GHS 4.00. Local drinks [traditional SBs] are better, though they take time to prepare” (Person 5, Male, Oyarifa).

“Sometimes you just crave a drink. If you have GHS 4.00, Bel Cola becomes an easier option.” (Person 5, Male, Oyarifa).

Conversely, some participants attributed a reduction in how often they purchased commercial SBs to what they perceived as tax-driven price increases:

“The price has indeed gone up. Before, if you had GHS 5.00, you could even get two, but now one is GHS 3.50 or GHS 4.00. So, we just buy one once in a while” (Person 6, Female, Kweiman).

“The price has changed…at first if you had GHS 2.50 pesewas, or GHS2.00, you could get one Coke and drink to calm your heart. But now its not like that…they have increased it, so we don’t often buy it, it’s now expensive” (Person 7, Female, Kweiman).

The survey quantified participants’ stated intentions regarding consumption change. The FGD accounts extended this by illustrating how purchasing power, taste preferences, and habitual consumption shaped responses in practice.

A summary of key findings is available in an infographic presentation in Supplementary File S5.

## 4. Discussion

To the best of our knowledge, this is the first community-based mixed-methods study to explore the factors influencing water and SB purchasing decisions and consumption behaviours among low-income households in peri-urban Accra following the implementation of Ghana’s beverage-tax policy. We used survey data and FGD accounts to examine water insecurity, purchasing decisions, and perceptions of the beverage tax. The convergent parallel design allowed us to quantify the prevalence and patterns of water insecurity and SB purchasing through the survey, while the FGD accounts provided depth on the motivations, contextual constraints, and lived experiences that the survey alone could not capture, together producing a more complete account than either strand would have yielded independently. Our findings indicate that awareness of the beverage taxation was limited, SBs remained highly patronised despite perceived price increases, and structural water insecurity constrained healthy hydration choices across all four communities. The findings suggest that fiscal measures alone are unlikely to support meaningful changes in beverage purchasing patterns in settings where water insecurity, limited tax awareness, and ingrained consumption habits persist. The following paragraphs discuss these findings in turn.

### 4.1. Water Insecurity and Sachet Water Dependence

Nearly 60% of participants reported experiencing water insecurity in the four weeks prior to the study. This finding is consistent with existing evidence that water infrastructure in peri-urban areas is often inadequately catered for by formal municipal planning [38]. Participants reported that inconsistent municipal supply and high water costs made them to draw on multiple sources (sachet, borehole, stored, and piped water) for drinking and domestic purposes. Participants linked these practices with an increased risk of water-borne infections and dehydration. Consistent with earlier work, this study observed households rationing sachet water for drinking while using cheaper alternatives for cooking and cleaning, reflecting a pragmatic approach to resource management [39].

Sachet water emerged as the dominant drinking source. Participants preferred sachet water for its perceived affordability, while bottled water, though viewed as safer, was considered too costly for regular household use. These findings are consistent with previous studies showing sachet water is favoured in low-income areas for its affordability and wide availability [40,41,42,43]. The FGD accounts added depth, revealing that brand loyalty, prior illness experience, and vendor trust further shaped purchasing decisions. Despite growing concerns about plastic pollution and variable product quality, sachet water was exempted from the beverage tax to make it affordable for low-income households [44]. Our findings suggest that taxing bottled water and SB equally is unlikely to steer consumers towards healthier options. Tax design and water infrastructure investment need to be addressed together.

### 4.2. Drivers of SB Consumption

The majority of households (69.8%) reported consuming commercial SBs daily or near-daily. Taste was the most cited purchasing consideration (62.8%), followed by convenience (46.5%), perceived safety and health (34.9%), availability (32.6%), and price (20.9%). Taste, particularly the perceived sweetness of carbonated drinks, was consistently prioritised over price and health concerns. Participants reported craving SBs and described this as a key consideration in unplanned purchases. A comparable pattern was reported in Mexico, where adolescents continued purchasing SBs despite tax-related price increases because of strong taste preferences and cravings [45].

Convenience was also central to SB purchasing. SBs were easily accessible through informal vendors and tricycle traders, known locally as “aboboyaa”, operating within residential areas. Purchases were described as quick and effortless, particularly for households with limited transportation. Informal vendors often operated with lower overhead costs and, in some cases, outside formal tax systems. This made SBs more attractively priced than in formal retail outlets, limiting the reach of the tax among price-sensitive consumers [15,46].

Purchasing power was an additional consideration. Limited household budgets were associated with switching between commercial and traditional SBs rather than reducing overall consumption. Regular SB consumption in the evenings after meals was described as a common household social norm. Evidence from Mexico suggests that SB taxes are associated with reduced purchases, particularly among low-income households who tend to be more sensitive to price changes [45]. The 2023 Tax Amendment extended the tax to fruit and vegetable juices. However, juices remained considerably more expensive than commercial SBs and were not perceived as a viable alternative by participants. These findings suggest that prices changes alone may not be sufficient to shift SB purchasing patterns in low-income peri-urban settings. Fiscal policy needs to address the relative prices of SBs and healthier alternatives, and the ease of access to affordable SBs through informal markets.

### 4.3. Children’s Preferences and Household SB Consumption

Children also emerged as an important factor associated with SB consumption. Participants reported buying SBs primarily to meet children’s requests and brand preferences, often overriding their own purchasing choices. When prices rose, parents switched to cheaper brands or traditional alternatives rather than reducing consumption. This pattern suggests that children’s preferences were associated with maintaining or increasing household SB purchasing regardless of price change [47,48]. Research links excessive SB consumption in children to insulin resistance and other metabolic effects [49,50]. These findings suggest that reducing household SB consumption requires addressing children’s preferences directly. Fiscal policy needs to be complemented by strategies targeting the school and home food environment.

### 4.4. Nutritional Implications of Substitution with Local Beverages

The nutritional implications of substitution with traditional or homemade SBs remain uncertain. Traditional SBs such as sobolo (hibiscus), asana (malted maize), zomkom (rice-based), and lamugin (millet-based) are typically prepared with added sugar. Their per-serving sugar content may rival or exceed that of commercial SBs depending on household preparation practices [15,51], though some of the local alternatives may carry nutritional benefits. For example, hibiscus-based sobolo contains polyphenols and cereal-based SBs may provide fibre. However, these traditional SBs are not categorically healthier than commercial SBs. We did not measure the sugar content of homemade beverages in this study. We therefore cannot determine whether switching to local alternatives reduces, maintains, or increases overall sugar intake. Researchers should compare the nutritional profiles of taxed commercial SBs and untaxed local alternatives in low-income peri-urban settings. Such findings would inform both fiscal and public health education policy.

### 4.5. Tax Awareness, Perceptions and Health Risk Perceptions

Although just over half of participants (55.8%) reported some awareness of the beverage tax, awareness was largely superficial. Most participants conflated general price increases driven by inflation with those specifically attributable to the tax. This suggests low policy visibility, consistent with previous research in Ghana [52]. Many believed the tax was levied twice, once on producers and again on consumers. This created a perceived sense of double burden. Where policy visibility is low, the assumption that fiscal measures will discourage SB purchases may not hold [53]. SBs remained highly patronised despite perceived price increases [54]. More participants opposed the taxation of bottled water than the taxation of SBs (62.8% vs. 46.5%). They perceived water as a fundamental necessity that should not be taxed. Consistent with a previous study in Ghana, participants perceived the beverage tax as revenue-driven rather than a public health measure [55].

Participants associated this perception with a lack of transparency about how tax revenue was used. Notably, 79.1% of participants indicated they would support the SB tax if revenue was earmarked for improving water infrastructure. Evidence from South Africa and Mexico indicates that public support for health-related taxes is stronger when citizens understand the rationale and observe revenues invested in visible public goods [14,56]. These findings suggest that strengthening tax communication and making revenue use transparent are necessary for building public legitimacy for the SB tax.

The findings also show that participants readily identified carbonated soft drinks as SBs. They widely recognised the association between SB consumption and diabetes risk (83.7%). However, awareness of links to obesity (46.5%) and cancer (19%) was substantially lower. Many underestimated the sugar content of energy drinks, fruit juices, sports drinks, and traditional or homemade beverages. We term this pattern “selective risk perception”. It may limit behaviour change. Participants poorly understood the concept of “hidden sugars”, sugars present in beverages not commonly perceived as sweet. This is particularly concerning. Participants who substituted commercial SBs with traditional homemade beverages may not have recognised these alternatives as contributing to their overall sugar intake. Although we explored participants’ knowledge, attitudes, and practices related to SB consumption, we did not directly probe their prior exposure to formal health education. This is a further limitation of the present study. Previous research in South Africa found that fiscal measures were associated with reduced SB intake even without directly addressing awareness [57].

Nonetheless, our findings underscore the need for strengthened public health education. Such education should clarify the concept of “hidden sugars”. It should also expand understanding of SB-related health risks beyond diabetes to include obesity and metabolic disease. It should improve communication about the purpose and benefits of the SB tax. The results also show that knowledge alone does not change behaviour. Even though many participants had heard that SBs were associated with diabetes, this awareness did not translate into reduced purchasing [58,59]. Combining fiscal policy with targeted nutrition education and, where feasible, behavioural-psychology-informed interventions may therefore be necessary to translate awareness into behaviour change in low-income peri-urban settings. These findings point to the need for targeted public health education as an essential complement to fiscal policy.

### 4.6. Integrating the Findings

Our findings point to interrelated structural, behavioural, and policy-level constraints that may limit the effectiveness of Ghana’s current beverage-tax design. In settings where safe water is scarce and sachet water is the main drinking source, taxing bottled water and SBs equally may offer households limited incentive to choose safer hydration options. We also found that SB consumption was associated with taste preferences, convenience, purchasing power, children’s preferences, and ingrained habits. Price changes alone may not readily address these factors. Participants reported limited tax awareness, perceptions of unfairness, and selective health risk perception. These were all linked to lower responsiveness to the tax. These patterns are consistent with the Theory of Planned Behaviour, which distinguishes between awareness and behavioural intention [58,59]. They are also consistent with the Stimulus-Response Model, which suggests that internal consumer factors may interact with external fiscal stimuli in shaping purchasing behaviour [19]. We suggest that addressing these constraints may require a coordinated package of fiscal, infrastructure, educational, and regulatory measures.

### 4.7. Policy Implications

The recommendations below combine direct findings from this study (participant perceptions, reported behaviours, and expressed support for revenue earmarking) with established evidence from comparable settings (Mexico and South Africa) [14,57]. Although our findings are drawn from four peri-urban communities in Accra, the structural conditions they describe—limited and inconsistent access to potable water, reliance on sachet water as the primary drinking source, price-sensitive purchasing in informal markets, and uneven awareness of SB-related health risks—are documented across West Africa and the wider WHO African Region [5,6]. Five recommendations follow from our analysis.

First, Ghana should refine its beverage tax. The current 20% ad valorem rate applies equally to bottled water and SBs. This undermines the public health intent of taxation by removing the price wedge that would steer consumption toward water. The rate on SBs should be increased and bottled water should be exempted from the tax, so that price signals favour the healthier hydration option. This recommendation is consistent with WHO guidance on SB taxation [9] and with evidence from South Africa and Mexico that meaningful price differentials are necessary for fiscal policy to influence purchasing in low-income populations [14,57].

Second, beverage-tax revenue should be earmarked for potable water infrastructure. Our finding that 79.1% of participants would support an SB tax if revenue were used to improve water access indicates a feasible path to building public legitimacy for the tax. Visible reinvestment in peri-urban water supply would also address the structural barrier that currently drives substitution toward cheaper, unregulated alternatives.

Third, tax communication and public health education should be strengthened in parallel. Targeted campaigns are needed to make the rationale and use of the tax visible to consumers, broaden risk awareness beyond diabetes to include obesity and other SB-related conditions, and clarify hidden sugars in less obvious products. This is particularly important for energy drinks, fruit juices, sports drinks, and traditional or homemade beverages, the sugar content of which our participants widely underestimated.

Fourth, regulatory oversight of sachet water should be strengthened alongside investment in piped supply. Sachet water now serves as the primary drinking source for the majority of low-income peri-urban households, yet its quality is variable and its production is incompletely regulated [44]. Nine of the 43 surveyed households (21%) reported episodes of stomach pain or diarrhoea after drinking sachet water (Supplementary File S4: Table S2), supporting the need to strengthen oversight of production and vendor practices to safeguard the population that depends on it. Future research should investigate the use of environmentally friendly and less expensive and more hygienic packaging, such as water jars or dispenser bottles, as alternatives to sachet water.

Fifth, validated household water insecurity tools, such as the HWISE-12 used in this study, should be incorporated into routine national surveys in Ghana and across the WHO African Region. Routine measurement would support equity-targeted policy design and allow tax and infrastructure interventions to be evaluated against population-level water-security outcomes.

These five recommendations point to a coordinated package of fiscal, regulatory, infrastructure, and educational measures rather than isolated interventions. Their relevance extends beyond Ghana to other West African countries where SB sales are rising, peri-urban water access is constrained, and tax architectures are under review [5,6].

### 4.8. Methodological Considerations

This study has five limitations. First, its small sample size (*n* = 43) and focus on four specific communities make the findings context-bound and not statistically generalizable to the wider Ghanaian population. Second, the reliance on self-reported data for water insecurity and purchasing habits may introduce recall and social desirability biases; thus, the study findings should be interpreted with caution. Third, although our theoretical framework drew on the Theory of Planned Behaviour and our findings engage constructs central to nutrition psychology and consumer behaviour including taste preferences, cravings, brand familiarity, social norms, and children’s influence on household purchasing, the study did not include validated psychological measures of constructs such as impulsivity, food-related self-regulation, or risk perception. Future research would benefit from incorporating such measures to complement the behavioural drivers identified here. Consequently, participants’ stated intentions to switch beverages due to the tax may not accurately predict actual behavioural shifts, particularly when ingrained taste preferences and cravings persist. Fourth, our FGD guide did not directly probe participants’ prior exposure to formal nutrition or hydration education programmes, and we note this as a limitation and a gap for future research. Fifth, although we collected data across four peri-urban communities to strengthen transferability, the findings should not be overstated beyond the study context. They are best understood as exploratory evidence that points to patterns warranting further investigation in broader populations.

However, several steps were taken to enhance the trustworthiness and rigour of this study. First, we collected data across four peri-urban sites representing diverse socio-economic contexts. This supported the transferability of the findings within the study context. Second, we used a convergent parallel mixed-methods design. We combined quantitative household survey data and qualitative FGD accounts. This allowed triangulation and provided a more complete understanding of water and SB purchasing decisions and behaviours than either strand alone. Third, we pilot-tested both the survey questionnaire and FGD guide in a community with similar characteristics before fieldwork. We revised both instruments to improve clarity, cultural relevance, and linguistic appropriateness.

To ensure credibility, we audio-recorded all FGDs and transcribed them verbatim in Twi or Ga. We subsequently translated all transcripts into English. Two researchers independently coded the data. Where disagreements arose, we resolved them through discussion and consensus. This team-based approach reduced potential bias and strengthened reliability. We used a hybrid thematic analysis approach combining deductive coding, guided by study objectives and existing literature, and inductive coding emerging from participant narratives. This combination strengthened the robustness of theme development. We assessed data saturation during transcript review and team debriefing. By the fourth FGD, no new major codes or themes were emerging. This supported our judgement that thematic saturation had been reached. We strengthened the rigour and transparency of the study through detailed documentation of study procedures, iterative refinement of data collection tools and reflexive team discussions to address potential research bias. We adhered to the COREQ 32-item transparent reporting of qualitative research procedures (Supplementary File S2).

## 5. Conclusions

Low-income households in peri-urban Accra reported making beverage choices largely based on price, perceived safety, taste, and availability. Sachet water was the dominant drinking source, preferred for its affordability and perceived safety. SB intake remains high, linked to perceived taste, convenience, and children’s preferences.

From a public health perspective, our findings suggest that health awareness is secondary to economic and sensory drivers. While awareness of diabetes risk was high, a “selective risk perception” persists regarding obesity, cancer, and other SB-related conditions, and the sugar content of energy drinks, fruit juices, and traditional beverages was widely underestimated. Furthermore, the lack of safe, untaxed water alternatives creates a structural barrier to healthy hydration, so that price-sensitive consumers substitute taxed SBs with cheaper, unregulated local SBs rather than shifting toward water. Without parallel investment in potable water access and targeted education on “hidden sugars” and the broader spectrum of SB-related health risks, the current tax design risks displacing consumption toward less regulated alternatives without reducing overall sugar intake with adverse implications for the diet-related non-communicable disease burden in low-income peri-urban populations. Combining improved access to safe, affordable water with targeted public health education is therefore essential for translating fiscal policy into measurable health gains.

Self-reported purchasing patterns and FGD accounts in these communities suggest that the 2023 beverage tax has, to date, had a limited apparent effect on overall SB consumption, although a study of this design and sample size cannot answer this question definitively. We also note that our FGD guide did not probe participants’ prior exposure to formal water consumption or nutrition education. Future research should examine whether targeted water and nutrition education, delivered alongside improved water access, is associated with healthier hydration choices and reduced SB consumption in low-income peri-urban settings. Taxing bottled water alongside SB risks could undermine access to healthier alternatives in contexts where the supply of potable water is limited. To better align with health and equity goals, the government should consider increasing the tax on SBs to create a larger price wedge between SB products and bottled water. Additionally, there is a critical need to earmark the resultant tax revenue toward improving potable water infrastructure. Improving access to safe, affordable water in communities and schools, alongside targeted health education on hidden sugars, may be necessary for promoting healthy hydration and supporting sustained reductions in SB consumption.

## Figures and Tables

**Table 1:** Socio-demographic characteristics of study participants, March–April 2025.

Attribute	Category	Survey (* *n* = 43)	** FGDs (*n* = 36)
Sex			
	Female	27 (62.8%)	24 (66.7%)
	Male	16 (37.2%)	12 (33.3%)
Age Category			
	18–35 yrs	23 (53.5%)	20 (55.6%)
	36–64 yrs	20 (46.5%)	16 (44.4%)
Ethnic Group			
	Ga/Adangbe	24 (55.8%)	20 (55.6%)
	Akan/Fante	7 (16.3%)	6 (16.7%)
	Northern	6 (14.0%)	5 (13.9%)
	Ewe	5 (11.6%)	4 (11.1%)
	Wassa	1 (2.3%)	1 (2.8%)
Religion			
	Christianity	38 (88.4%)	32 (88.9%)
	Islam	4 (9.3%)	3 (8.3%)
	No religion	1 (2.3%)	1 (2.8%)
Occupation			
	Unemployed	5 (11.6%)	4 (11.1%)
	Self-employed (traders, artisans, etc.)	36 (83.7%)	30 (69.8%)
	Formal sector (e.g., teacher, etc.)	2 (4.7%)	2 (5.6%)
Household Size			
	≤3 members	11 (25.6%)	9 (25.0%)
	4 members	11 (25.6%)	9 (25.0%)
	5–10 members	21 (48.8%)	18 (50.0%)
Educational Level			
	None	8 (18.6%)	6 (16.7%)
	Primary	1 (2.3%)	1 (2.8%)
	Junior high school	28 (65.1%)	24 (66.7%)
	Senior high school	5 (11.6%)	4 (11.1%)
	Tertiary	1 (2.3%)	1 (2.8%)
Relationship to Household Head			
	Self	23 (53.5%)	20 (55.6%)
	Spouse	20 (46.5%)	16 (44.4%)
Wealth Index			
	Lowest	11 (25.6%)	9 (25.0%)
	Moderate	11 (25.6%)	9 (25.0%)
	High	12 (27.9%)	10 (27.8%)
	Highest	9 (20.9%)	8 (22.2%)

* *n* = sample size. ** FGD is Focus Group Discussion.

**Table 2:** Household water insecurity experiences (HWISE) and water and SB purchasing behaviour among study participants, March–April 2025 (*n* = 43).

Attributes	Categories	Frequency (*n*)	Percent (%)
Household Water Insecurity Experiences (HWISE) *			
	No-to-marginal	17 (39.5%)	-
	Low	9 (20.9%)	-
	Moderate	10 (23.3%)	-
	High	7 (16.3%)	-
Main Drinking Water Source			
	Sachet only	33	76.7%
	Sachet and other sources **	9	20.9%
	Rainwater only	1	2.3%
Water Purchase Frequency			
	Daily/3–4× weekly	25	58.1%
	Weekly/2× weekly	18	41.9%
Sachet Water Quantity Purchased (per week)			
	<1 bag ***	7	16.3%
	1 bag	8	18.6%
	2 bags	6	14.0%
	≥3 bags	22	51.2%
Weekly Sachet Water Spending (GHS ****)			
	<10	8	18.6%
	10–20	7	16.3%
	21–50	13	30.2%
	>50	15	34.9%
Beverage Type Purchased			
	Commercial SBs only	30	69.8%
	Both commercial SBs and traditional/local/homemade SBs	5	11.6%
	Traditional/local/homemade SB	3	7.0%
	Mixing SBs with alcohol	5	11.6%
Beverage Purchase Frequency			
	Low (monthly/rarely)	5	11.6%
	Medium (weekly/2× weekly)	10	23.3%
	High (daily/3–4× weekly)	28	65.1%
Units of Beverages Per Week			
	Rarely	1	2.3%
	1–2 bottles *****	12	27.9%
	3–5 bottles	13	30.2%
	6–10 bottles	9	20.9%
	>10 bottles	8	18.6%
Weekly Beverage Spending (GHS)			
	<10	9	20.9%
	10–30	10	23.3%
	31–50	8	18.6%
	>50	15	34.9%

* HWISE Score Range: No-to-marginal (0–3), low (4–11), moderate (12–23) and high (24–36). ** Other sources are borehole, wells, piped or tap water. *** A bag or pack of sachet water contains 30 sachets and each sachet typically has 500 mL of water, so a pack or bag of 30 sachets altogether gives 15 litres of water, **** GHS—Ghana Cedis. ***** A bottle of beverage is equivalent to 350 mL.

**Table 3:** Awareness and perceptions of beverage-tax policy among surveyed participants, March–April 2025 (*n* = 43).

Attributes	Frequency (*n* *)	Percent (%)
Awareness of beverage-tax policy		
Yes (Aware of the tax)	24	55.8%
No (Unaware of the tax)	19	44.2%
Attitude toward SB tax		
Oppose	20	46.5%
Support	23	53.5%
Attitude towards bottled water tax		
Oppose	27	62.8%
Support	16	34.9%
Attitude towards use of SB tax revenue for improving water infrastructure		
Oppose	9	20.9%
Support	34	79.1%
Potential changes in purchasing behaviour		
Continue to consume same drinks		
Yes	30	69.8%
No	13	30.2%
Cut back on sweet beverage consumption		
Yes	19	44.2%
No	24	55.8%
Switch to untaxed drinks		
Yes	20	46.5%
No	23	53.5%

* *n* = sample size.

## Data Availability

All data generated or analysed during this study are included in this published article.

## Supplementary Materials

The following supporting information can be downloaded at https://www.mdpi.com/article/10.3390/ijerph23060799/s1, Supplementary File S1: Flowchart showing the methodology applied in the study; Supplementary File S2: Additional Results Part 1; Supplementary File S3: Consolidated Criteria for Reporting Qualitative Research (COREQ), 32-item Checklist for Interviews and Focus groups; Supplementary File S4: Additional Results Part 2; Supplementary File S5: Infographics of summary of key findings.

## Abbreviations

The following abbreviations are used in this manuscript:

SBs	Sweet Beverages
NCDs	Non-Communicable Diseases
WHO	World Health Organization
IMF	International Monetary Fund
TPB	Theory of Planned Behaviour
FGD	Focus Group Discussion
ECBAS	Ethics Committee for Basic and Applied Sciences
HWISE	Household Water Insecurity Experiences
MPI	Multidimensional Poverty Index
COREQ	Consolidated Criteria for Reporting Qualitative Research
PCA	Principal Component Analysis
