# Supplementary material for: Factors Influencing Water and Sweet Beverage Purchasing Decisions and Behaviours Among Low-Income Households in Four Peri-Urban Communities in Accra: An Exploratory Study
Source: Int J Environ Res Public Health. 2026 Jun 15;23(6):799. doi: 10.3390/ijerph23060799 (PMC13299423; doi:10.3390/ijerph23060799)
Supplement: Supplementary file 1 [file ijerph-23-00799-s001.zip › Supplementary File S2 Additional Method and Results Part 1.pdf]

Supplementary Files 2: Additional Method and Results

Supplementary File S2:

Supplementary File S2 Methods M1

Multiple Poverty Index (MPI) among surveyed participants residing in Oyarifa, Teiman, Kweiman, and Danfa

We adapted the Multidimensional Poverty Index (MPI) using the Alkire–Foster method to assess household deprivation across multiple dimensions of wellbeing [31]. Three dimensions of wellbeing were considered: health, education, and housing and living conditions. Eleven indicators were used to construct the MPI, consistent with previous studies in similar contexts [31]. For each indicator, households were first assessed against a predefined deprivation cut-off (e.g., use of unimproved cooking fuel, lack of improved water source, absence of household sanitation facility, lack of electricity, overcrowding, non-ownership of selected household assets, non-enrolment in the National Health Insurance Scheme, school non-attendance, low educational attainment, and school lag). Households meeting the deprivation criterion for an indicator were coded as deprived (1), while those not deprived were coded as non-deprived (0). Indicators were grouped under their respective dimensions, and equal weights were assigned to each indicator within a dimension. The weighted deprivation scores for all indicators were then summed for each household to generate a household-level MPI score, ranging from 0 to 1. A household was classified as multi-dimensionally poor if it was deprived in at least one-third (≥33%) of the weighted indicators, in line with the Alkire–Foster poverty cut-off [1]. To enable comparison across study sites, community-level MPI scores were computed by aggregating household MPI scores within each community, reflecting the intensity of deprivation across the four peri-urban settings. The MPI was used alongside the PCA-based wealth index to capture non-monetary aspects of poverty, thereby providing a more comprehensive picture of household living conditions and socioeconomic vulnerability (

Additional Results

Supplementary File S2: Multiple Poverty Index (MPI) among surveyed participants residing in Oyarifa, Teiman, Kweiman, and Danfa

Supplementary File S2: Tables S1-S3 and Figure S1 illustrate the nature of poverty in these settings. While only about one in ten households met the criteria for multidimensional poverty, the intensity of deprivation among those classified as poor was substantial, with households simultaneously deprived in sanitation, cooking fuel, and schooling. Danfa emerged as the most deprived site, with the highest MPI score, while Oyarifa and Teiman displayed slightly lower scores. This indicates that although income poverty may not appear widespread, multiple overlapping deprivations in housing, education, and basic services remain significant. These are mean proportions of households deprived in each community by MPI indicator.

Supplementary File S2: Table S1: Summary of Multiple Poverty Index (MPI) among surveyed participants, March-April, 2025

**Indicator**	**Value**
**Total Households**	43
**# MPI Poor**	5
**# Not Poor**	38
**Headcount (H)**	11.6%
**Intensity (A)**	41.1%
MPI Score (H × A)	**0.048**

Supplementary File S2: Table S2: MPI Summary by Community (Oyarifa, Teiman, Kweiman, and Danfa) in the Ga East Municipality, March-April, 2025

**Community**	**Total HHs**	**# MPI Poor**	**Headcount (H)**	**Intensity (A)**	**MPI Score**
**Danfa**	11	2	18.2%	41.4%	0.075
**Kweiman**	10	1	10.0%	45.0%	0.045
**Oyarifa**	11	1	9.1%	38.7%	0.035
**Teiman**	11	1	9.1%	39.2%	0.036

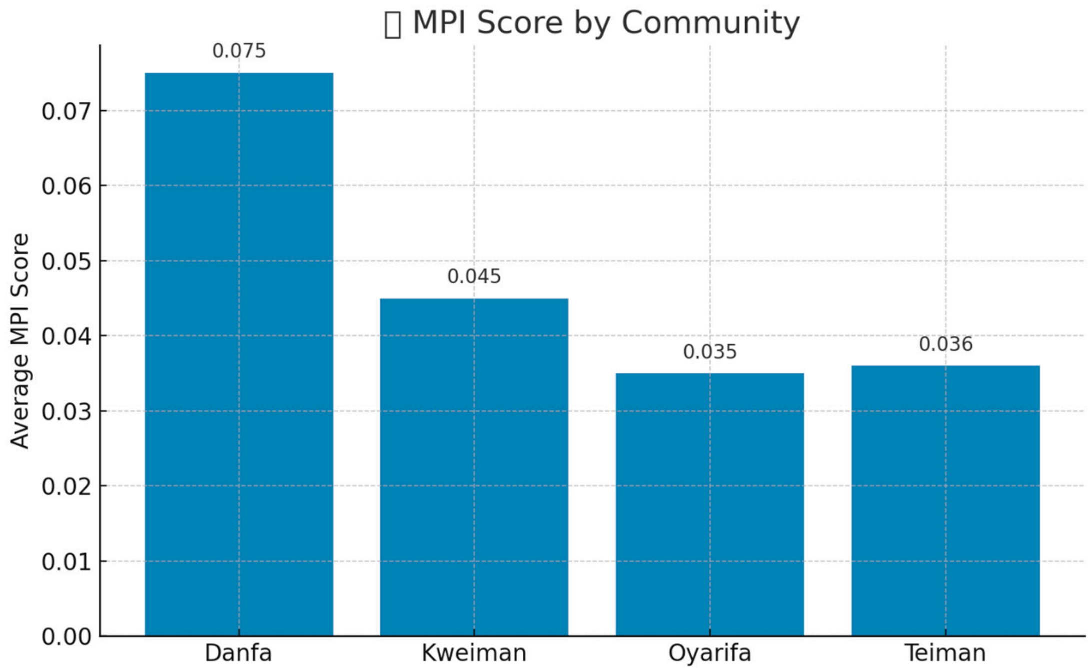

Supplementary File S2: Figure S1: MPI Score by community, March-April, 2025

Supplementary File S2: Table S3: Proportion of Households Deprived by wellbeing indicators among surveyed participants, March-April, 2025

Indicators of Wellbeing	**Community**			
	**Danfa**	**Kweiman**	**Oyarifa**	**Teiman**
Cooking	27.0%	20.0%	18.0%	18.0%
Water	18.0%	30.0%	27.0%	27.0%
Sanitation	45.0%	40.0%	36.0%	36.0%
Housing	36.0%	30.0%	27.0%	18.0%
Electricity	9.0%	20.0%	9.0%	9.0%
Overcrowding	18.0%	10.0%	18.0%	27.0%
Assets	27.0%	30.0%	18.0%	18.0%
NHIS*	18.0%	10.0%	18.0%	18.0%
School Attendance	18.0%	20.0%	18.0%	9.0%
School Attainment	27.0%	10.0%	18.0%	27.0%
School Lag	9.0%	10.0%	18.0%	18.0%

*NHIS stands for National Health Insurance Scheme

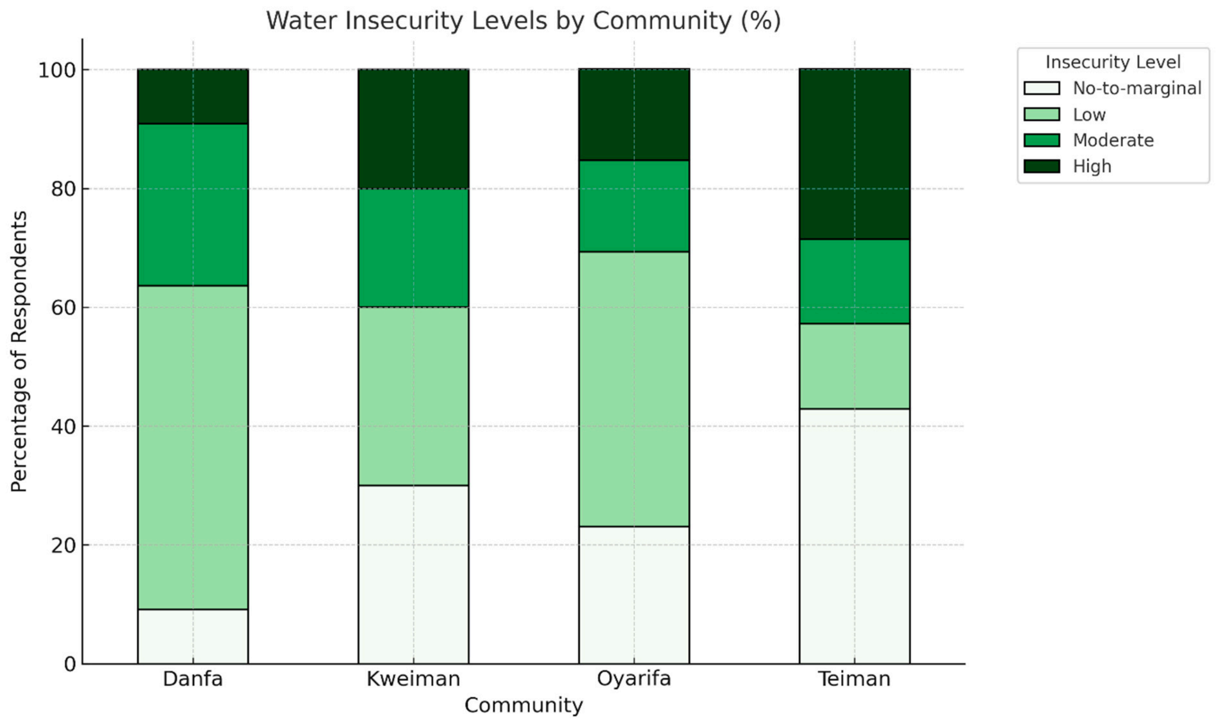

Supplementary File S2: Figure S2: Household water insecurity (HWiSE-12) by community, March-April, 2025
