# Supplementary material for: Factors Influencing Water and Sweet Beverage Purchasing Decisions and Behaviours Among Low-Income Households in Four Peri-Urban Communities in Accra: An Exploratory Study
Source: Int J Environ Res Public Health. 2026 Jun 15;23(6):799. doi: 10.3390/ijerph23060799 (PMC13299423; doi:10.3390/ijerph23060799)
Supplement: Supplementary file 1 [file ijerph-23-00799-s001.zip › Supplementary File S3 COREQ.pdf]

**Supplementary File 3: Consolidated Criteria for Reporting Qualitative Research (COREQ), 32-item Checklist for Interviews and Focus groups**

| Section/Topic | Item No | Checklist item | Reported on page No |
|---|---|---|---|
| **Domain 1: Research team and reflexivity** | | | |
| **Personal Characteristics** | | | |
| *Interviewer/facilitator* | 1 | Which author/s conducted the interview or focus group/Interviewer/facilitator | Christopher Delali Amegah(CDA) facilitated the FGD |
| *Credentials* | 2 | What were the researcher's credentials? E.g. PhD, MD | MPhil Candidate (Nutrition, University of Ghana |
| *Occupation* | 3 | What was their occupation at the time of the study? | Graduate student researcher (MPhil Nutrition) |
| *Gender* | 4 | Was the researcher male or female? | Male |
| *Experience and training* | 5 | What experience or training did the researcher have? Relationship with participants | Researchers received training in qualitative research, data collection, and facilitation of FGDs; supervised by experienced faculty (Prof. Seth Adu-Afarwuah, Dr. Gloria Odei Obeng-Amoako |
| **Relationship with participants** | | | |
| *Relationship established* | 6 | Was a relationship established prior to study commencement? | Yes, rapport was built during community entry and quantitative data collection |
| *Participant knowledge of the interviewer* | 7 | What did the participants know about the researcher? e.g. personal goals, reasons for doing the research | Participants knew researcher was from University of Ghana, conducting research on water and beverages, supported by supervisors |
| *Interviewer characteristics* | 8 | What characteristics were reported about the interviewer/facilitator? e.g. Bias, assumptions, reasons and interests in the research topic | Participants saw interviewer had reasons and interests in the research topic |
| **Domain 2: study design** | | | |
| Theoretical framework | | | |
| *Methodological orientation and Theory* | 9 | What methodological orientation was stated to underpin the study? e.g. grounded theory, discourse analysis, ethnography, phenomenology, content analysis | Theory of Planned Behaviour and Stimulus Response Model, hybrid inductive and deductive thematic analysis |

| Section/Topic | Item No | Checklist item | Reported on page No |
|---|---|---|---|
| | | | |
| **Participant selection** | | | |
| *Sampling* | 10 | How were participants selected? e.g. purposive, convenience, consecutive, snowball | Purposive sampling of households in four peri-urban communities, ensuring maximum variation |
| *Method of approach* | 11 | How were participants approached? e.g. face-to-face, telephone, mail, email | Participants were approached face-to-face in households and invited to participate |
| *Sample size* | 12 | How many participants were in the study? | 36 FGD participants (across 4 sites) |
| *Non-participation* | 13 | How many people refused to participate or dropped out? Reasons? | 7 parcipants were absent during FGD period |
| *Setting of data collection* | 14 | Where was the data collected? e.g. home, clinic, workplace | FGDs conducted in community venues (chief palaces, assembly halls, homes) |
| *Presence of non-participants* | 15 | Was anyone else present besides the participants and researchers? | Supervisors were present besides participants and research assistants in FGDs |
| *Description of sample* | 16 | What are the important characteristics of the sample? e.g. demographic data, date | Low-income adults (majority female, aged 18–64), mainly Ga/Adangbe, married/cohabiting, with large households and limited education |
| **Data collection** | | | |
| *Interview guide* | 17 | Were questions, prompts, guides provided by the authors? Was it pilot tested? | Yes, a semi-structured FGD guide was developed and pilot tested |
| *Repeat interviews* | 18 | Were repeat interviews carried out? If yes, how many? | No repeat interviews were carried out |
| *Audio/visual recording* | 19 | Did the research use audio or visual recording to collect the data? | Yes, FGDs were audio recorded by a recorder |
| *Field notes* | 20 | Were field notes made during and/or after the interview or focus group? | Yes, field notes were taken during and after discussions |

| Section/Topic | Item No | Checklist item | Reported on page No |
|---|---|---|---|
| *Duration* | 21 | What was the duration of the interviews or focus group? | FGDs lasted 1–2 hours (range: 1h15m to 2h05m) |
| *Data saturation* | 22 | Was data saturation discussed? | Yes, data saturation was discussed prior to data collection, during data collection, coding and confirmed across sites |
| *Transcripts returned* | 23 | Were transcripts returned to participants for comment and/or correction? | No, transcripts were not returned to participants |
| **Domain 3: analysis and findings**<br>**Data analysis** | | | |
| *Number of data coders* | 24 | How many data coders coded the data? | Two coders (CDA and MOM) independently coded transcripts with consensus reached |
| *Description of the coding tree* | 25 | Did authors provide a description of the coding tree? | Yes, a coding framework/codebook was developed in ATLAS.ti |
| *Derivation of themes* | 26 | Were themes identified in advance or derived from the data? | Themes were derived inductively from data, guided by study objectives and framework |
| *Software* | 27 | What software, if applicable, was used to manage the data? | ATLAS.ti software was used for coding and analysis |
| *Participant checking* | 28 | Did participants provide feedback on the findings? | No, participants did not provide feedback on findings |
| Reporting | | | |
| *Quotations presented* | 29 | Were participant quotations presented to illustrate the themes / findings? Was each quotation identified? e.g. participant number | Yes, verbatim quotes with participant ID (site, gender, number) were used in reporting |
| *Data and findings consistent* | 30 | Was there consistency between the data presented and the findings? | Yes, findings aligned with presented data and quotes |
| *Clarity of major themes* | 31 | Were major themes clearly presented in the findings? | Yes, major themes were clearly presented by study objectives |

| Section/Topic | Item No | Checklist item | Reported on page No |
|---|---|---|---|
| *Clarity of minor themes* | 32 | Is there a description of diverse cases or discussion of minor themes? | Yes, diverse and contrasting cases across sites were included |
