# Supplementary material for: Factors Influencing Water and Sweet Beverage Purchasing Decisions and Behaviours Among Low-Income Households in Four Peri-Urban Communities in Accra: An Exploratory Study
Source: Int J Environ Res Public Health. 2026 Jun 15;23(6):799. doi: 10.3390/ijerph23060799 (PMC13299423; doi:10.3390/ijerph23060799)
Supplement: Supplementary file 1 [file ijerph-23-00799-s001.zip › Supplementary File S4 Additional Results Part 2.pdf]

Supplementary File S4: Additional Results Part 2

Supplementary File S4: Table S1: Housing and sanitation characteristics among surveyed participants, March-April, 2025

Attributes	Frequency(n)	Percent(%)
Housing Characteristics		
**Wall Material**		
**Cement blocks/Bricks**	37	86.1%
**Wood/Thatch**	5	11.6%
**Other**	1	2.3%
Roofing Material		
**Metal sheet**	40	93.0%
**Mud/Earth**	1	2.3%
**Wood**	1	2.3%
**Other (e.g., concrete)**	1	2.3%
Floor Material		
**Cement/Concrete**	38	88.4%
**Vinyl/Tiles**	2	4.7%
**Wood**	3	7.0%
Toilet Facility Type		
**Shared flush toilet**	17	39.5%
**Public toilet**	14	32.6%
**Own flush toilet**	10	23.3%
**Pit latrine (shared)**	1	2.3%
**Other (e.g., open defecation)**	1	2.3%

Supplementary File S4: Table S2: Sachet Water Purchasing Behaviours (Per Week Estimates) among surveyed participants, March-April, 2025, (n=43)

Attributes	Frequency (n)	Percent
**Uses a Specific Brand Consistently**		
Yes	27	62.79%
No (buys different brands)	16	37.21%
**Reasons for Choosing Sachet Water Brand**	*(Multiple responses possible)*	
Health Considerations	13	30.23%
Availability	12	27.91%
Taste	6	13.95%
Convenience	2	4.65%
Health Considerations	13	30.23%
Other	7	16.28%
Other/mixed factors (e.g., public opinion, reputation)	7	16.28%
**Where Sachet Water is Purchased From**		
Tricycles	20	46.51%
Kiosks/stores in neighbourhood	18	41.86%
Delivered to home	4	9.30%
Other (e.g., water depot)	1	2.33%
**Reasons for Choosing That Source** *(multiple)*		
Availability	29	67.44%
Convenience	22	51.16%
Safety	20	46.51%
Taste	18	41.86%
Health Considerations	15	34.88%
Price	13	30.23%

**Attributes**	**Frequency (n)**	**Percent**
**Importance of Safety in Source Selection**		
Very Important	14	32.56%
Somewhat Important	18	41.86%
Not Important	11	25.58%
**Health Considerations in Water Selection** *(themes)*		
Concerned about germs, cleanliness, contamination	Majority	~70%+
No health concerns	Few (3 cases)	~7%
**Ever Felt Sick After Drinking Sachet Water**		
No	31	72.09%
Yes	9	20.93%
Not sure	3	6.98%
**If Yes, Type of Illness Experienced**	*(n=9)*	
Diarrhea	4	44.4%
Stomach pain	3	33.3%
Other (e.g., fever, vomiting)	2	22.2%

Supplementary File S4: Table S3: Bottled Water Purchasing Behaviours (Per Week Estimates) among surveyed participants, March-April, 2025, (n=43)

Attributes	Frequency (n)	Percent (%)
**Uses a Specific Brand**		
Yes	12	27.91%
No (buys different brands)	8	18.60%
Rarely / Not applicable	23	53.49%
**Common Bottled Water Brands Mentioned**		
Bel Aqua	9	20.93%
Others (Perla, Verna, Voltic)	3	6.98%
**Reasons for Brand Choice** *(multiple responses)*		
Taste	11	25.6%
Quality	10	23.3%
Price	7	16.3%
Health Consideration	6	14.0%
Availability	6	14.0%
Convenience	4	9.3%
**Where Bottled Water is Purchased**	*(among users)*	
Local store	11	25.58%
Street vendor / Supermarket	5	11.63%
Other (e.g., wholesale, gifted)	4	9.30%
Not applicable / Rare	23	53.49%
**Reasons for Choosing Purchase Source**	*(multiple responses)*	
Safety	17	39.53%
Packaging / Quality	14	32.56%
Brand / Reputation	12	27.91%
Social Perception	8	18.6%
Health Considerations	7	16.28%
Other(Proximity)	3	6.98%
**Believes Bottled Water is Safer Than Sachet Water**		

Attributes	Frequency (n)	Percent (%)
Yes	22	51.16%
No	10	23.26%
Unsure	8	18.60%
Least common response	3	6.98%
**Reasons for Considering Bottled Water "Safer"**		
Safety	17	39.53%
Packaging / Quality	14	32.56%
Brand / Reputation	12	27.91%
Social Perception	8	18.6%
Health Considerations	7	16.28%
Other	3	6.98%
**Has Recently Changed Bottled Water Purchases**		
No change	20	46.51%
Buys less	3	6.98%
Switched to other water sources	1	2.33%
Least reported / unclear reasons	19	44.19%
**Primary Reason for Change** *(if any)*		
Cost	2	4.65%
Taste preference	1	2.33%
Other (e.g., gift source, family needs)	4	9.30%

Supplementary File S4: Table S4: Commercial Beverage Purchasing Behaviours (Per Week) among surveyed participants, March-April, 2025, (n=43)

Attributes	Frequency (n)	Percent (%)
**Units of Beverages Purchased (Per Week)**		
1–2 bottles	12	27.91%
3–5 bottles	13	30.23%
6–10 bottles	9	20.93%
More than 10 bottles	8	18.60%
Rare/Unreported	1	2.33%
**Estimated Weekly Spending on Beverages**		
Less than GHS 10	9	20.93%
GHS 10–30	10	23.26%
GHS 31–50	8	18.60%
More than GHS 50	15	34.88%
Rare/Unreported	1	2.33%
**Reasons for Beverage Choice** *(multiple responses)*		
Taste	27	62.79%
Health Considerations	15	34.88%
Availability	14	32.56%
Safety	10	23.26%
Price	9	20.93%
Other (combined)	4	9.3%
**Sources of Beverages**		
Kiosks or local stores	34	79.07%
Supermarkets	2	4.65%
Street vendors / Other	6	13.95%
Least reported (home delivery / wholesale)	1	2.33%
**Reason Sources of Beverages**		
Availability	28	65.12%

**Attributes**	**Frequency (n)**	**Percent (%)**
Convenience	20	46.51%
Price	11	25.58%
Safety	10	23.26%
Health Considerations	6	13.95%
Other (combined)	4	9.3%
**Uses a Specific Brand**		
Yes	29	67.44%
No (buys different brands)	13	30.23%
Rare/Unreported	1	2.33%
**Popular Brands Mentioned**		
Bigoo, Bel Cola, Puka, Active Water	—	—
**Reasons for Choosing Brand** *(multiple responses)*		
Taste	18	41.86%
Availability	14	32.56%
Health Considerations	10	23.26%
Price	6	13.95%
Brand / Reputation	5	11.63%
Other (combined)	3	6.98%
**Average Daily Consumption** *(self-reported)*		
1–2 bottles	24	55.81%
3–5 bottles	14	32.56%
6 or more bottles	4	9.30%
Rare/Unreported	1	2.33%
**Primary Consumers in Household**	*(multiple)*	
Adults (18+)	Most common	—
Children and teenagers	Frequently cited	—
**Importance of Health in Beverage Choice**		

Attributes	Frequency (n)	Percent (%)
Very important	9	20.93%
Somewhat important	20	46.51%
Not important	13	30.23%
Rare/Unreported	1	2.33%
**Common Health Concerns Reported** *(multiple)*		
Too Much Sugar	20	46.5%
Artificial Ingredients	11	25.6%
Weight Gain / Stomach Problems	1	2.3%
No concerns	16	37.21%
**How They Ensure Beverage Safety**		
Trust the brand	23	53.49%
Read sugar content label	6	13.95%
Look for natural ingredients	4	9.30%
Family/friend advice	2	4.65%
Other (expiry date, preference, etc.)	1	2.33%
**Ever Felt Sick After Drinking a Beverage**		
No	36	83.72%
Yes	5	11.63%
Don't know	1	2.33%
Rare/Unreported	1	2.33%
**Exposure to Beverage Advertising**		
Yes	37	86.05%
No	4	9.30%
Rare/Unreported	2	4.65%
**Where Ads Were Seen** *(multiple sources)*		
Television	27	62.8%
Posters / Friends / Social Media	11	25.6%

Attributes	Frequency (n)	Percent (%)
Radio	5	11.6%
**Have Tried Beverages Based on Ads**		
Yes	7	16.28%
No	34	79.07%
Rare/Unreported	2	4.65%

Supplementary File S4: Table S5: Frequency of SB type was mentioned as when participants think of the term "sweetened beverages" among surveyed participants, March-April, 2025, (n=43)

**Attributes**	**Frequency (n)**	**Percent (%)**
Soft drinks	42	97.7%
Nectars or canned juices	13	30.2%
Flavored milk	12	27.9%
100% fruit juice	9	20.9%
Traditional drinks (e.g., Sobolo)	11	25.6%
Powdered drinks	5	11.6%
Energy drinks	5	11.6%
Unflavored milk	6	14.0%
Diet soda	2	4.7%
Sweetened iced tea	2	4.7%
Coffee/tea with sugar	2	4.7%
Sports drinks	2	4.7%
Bottled water (still/flavored)	2	4.7%
Cordials/concentrates	1	2.3%
Others (Local SBs)	1	2.3%
**Overall Knowledge Score**		
Mean (SD)	2.9 ± 2.2	
Median (Range)	2 (1–11)	
Knowledge Level		
Low Knowledge (0–2 correct items)	39	90.7%
Moderate Knowledge (3–5 items)	3	7.0%
High Knowledge (≥6 items)	1	2.3%

Supplementary File S4: Table S6: Perception of Sugar Content in Beverages among surveyed participants, March-April, 2025, (n=43)

Attributes	Frequency (n)	Percent (%)
Bottled water (still)		
Not sugary	43	100.0%
Somewhat sugary	-	-
Sugary	-	-
Bottled water (sparkling)		
Not sugary	26	60.5%
Somewhat sugary	-	-
Sugary	-	-
Bottled water (sparkling)		
Not sugary	-	-
Somewhat sugary	17	39.5%
Sugary	-	-
100% Fruit Juice		
Not sugary	8	18.6%
Somewhat sugary	27	62.8%
Sugary	8	18.6%
Flavored Milk		
Not sugary	8	18.6%
Somewhat sugary	26	60.5%
Sugary	9	20.9%
Soft Drinks		
Not Sugary	1	2.3%
Somewhat sugary	2	4.7%
Sugary	40	93.0%
Energy Drinks		
Not sugary	16	37.2%
Somewhat sugary	15	34.9%

**Attributes**	**Frequency (n)**	**Percent (%)**
Sugary	12	27.9%
Sweetened Iced Tea		
Not sugary	12	27.9%
Somewhat sugary	11	25.6%
Sugary	20	46.5%
Locally Made Drinks		
Not sugary	2	4.7%
Somewhat sugary	25	58.1%
Sugary	16	37.2%
Homemade Drinks/Juice		
Not Sugary	10	23.3%
Somewhat Sugary	28	65.1%
Sugary	5	11.6%
Sports/Isotonic Drinks		
Not sugary	24	55.8%
Somewhat sugary	13	30.2%
Sugary	6	14.0%
Perception Score		
Mean (SD)	—	0.46 ± 0.15
Median (IQR)	—	0.45 (0.35–0.55)
Min–Max	—	0.15 – 0.80
Perception Level		
Low Perception	3	7.0%
Moderate Perception	38	88.0%
High Perception	2	5.0%

Supplementary File S4: Table S7: Risk Perception of Health Effects of Sweetened Beverages among surveyed participants, March-April, 2025, (n=43)

**Attributes**	**Frequency**	**Percent (%)**
Perceived Risk of Health Conditions		
Diabetes		
No Risk	4	9.3%
A Little Risk	1	2.3%
Somewhat Risk	2	4.7%
A Lot of Risk	36	83.7%
High Blood Pressure		
No Risk	24	55.8%
A Little Risk	2	4.7%
Somewhat Risk	10	23.3%
A Lot of Risk	7	16.3%
Obesity		
No Risk	23	53.5%
A Little Risk	3	7.0%
Somewhat Risk	9	20.9%
A Lot of Risk	8	18.6%
Dental Problems		
No Risk	11	25.6%
A Little Risk	2	4.7%
Somewhat Risk	12	27.9%
A Lot of Risk	18	41.9%
Cancer		
No Risk	35	81.4%
A Little Risk	1	2.3%
Somewhat Risk	6	14.0%
A Lot of Risk	1	2.3%
**Risk Score**		
Mean (SD)	—	0.5 ± 0.2
Median (IQR)	—	0.5 (0.3–0.6)
Range (Min–Max)	—	0.2 – 0.7
Risk Perception Level		
Low Risk Perception	9	20.9%
Moderate Risk Perception	29	67.4%
High Risk Perception	5	11.6%
