# Supplementary figures and images for: Factors Influencing Water and Sweet Beverage Purchasing Decisions and Behaviours Among Low-Income Households in Four Peri-Urban Communities in Accra: An Exploratory Study
Source: Int J Environ Res Public Health. 2026 Jun 15;23(6):799. doi: 10.3390/ijerph23060799 (PMC13299423; doi:10.3390/ijerph23060799)

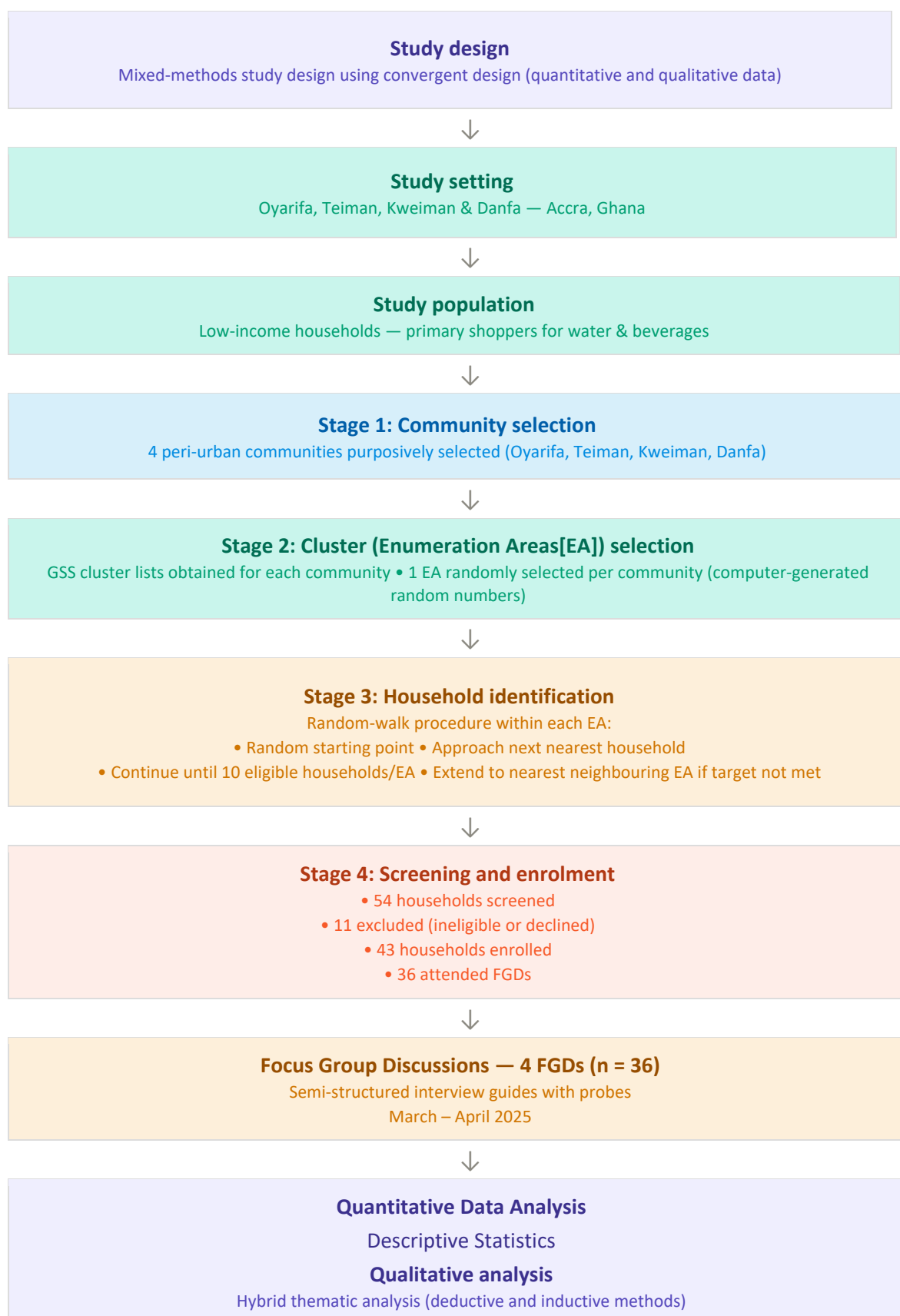

**Supplementary File S1: Figure S1: Flowchart showing the methodology applied in the study.**

Supplement: Supplementary file 1 [file ijerph-23-00799-s001.zip › Supplementary File S1 Flowchart on study methodologhy.pdf]

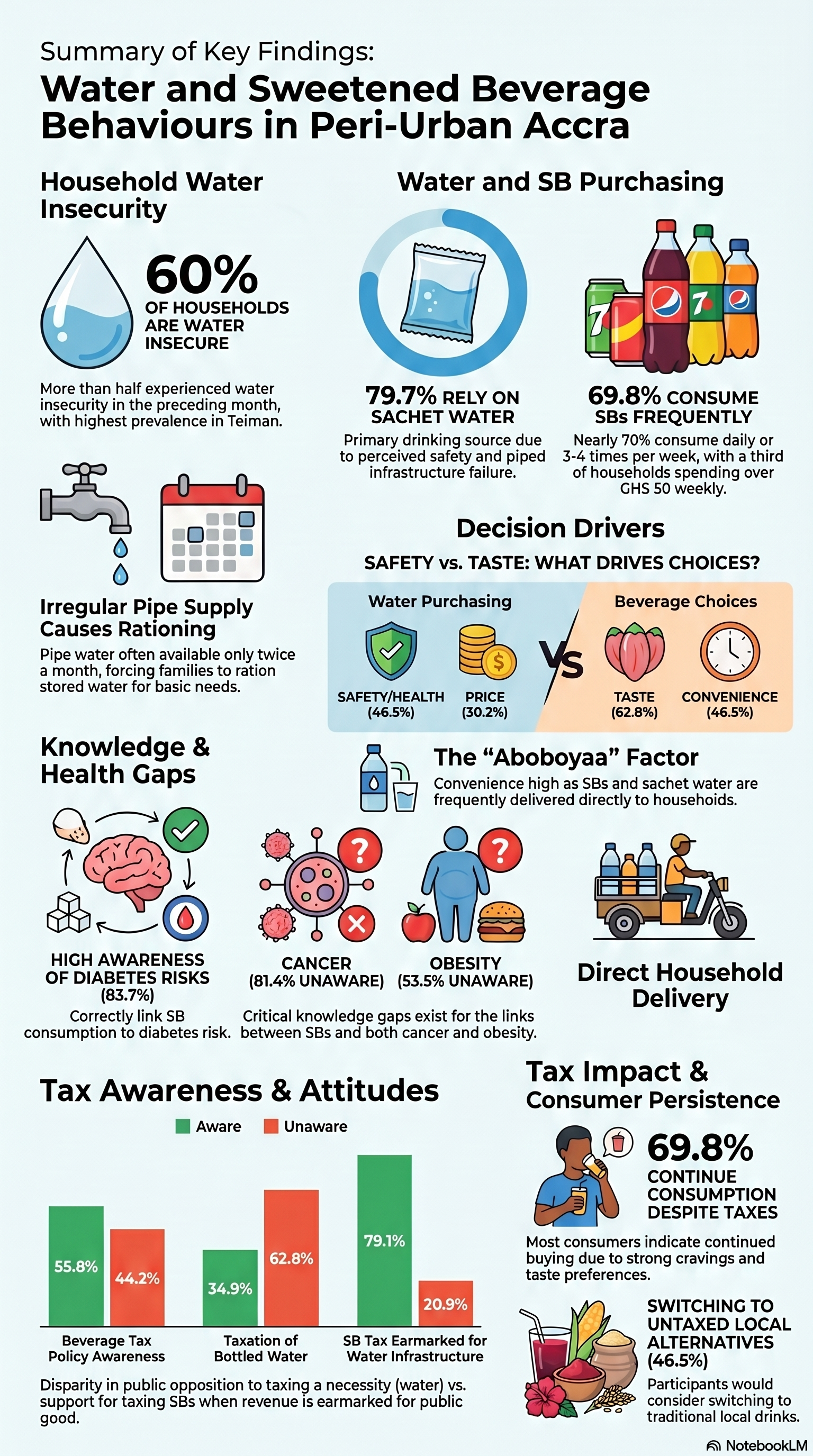

Supplement: Supplementary file 1 [file ijerph-23-00799-s001.zip › Supplementary File S5 Infographics of summary of key findings.png]
